# Sonochemical technologies in corrosion science and engineering: from laboratory studies to industrial perspectives^☆^

**DOI:** 10.1016/j.ultsonch.2026.108024

**Published:** 2026-08-25

**Authors:** Chandrabhan Verma, Anu Radha Pathania, Akram AlFantazi

**Affiliations:** a Department of Chemical and Petroleum Engineering, Khalifa University of Science and Technology, P.O. Box 127788, Abu Dhabi, United Arab Emirates; b Emirates Nuclear Technology Center (ENTC), Khalifa University of Science and Technology, Abu Dhabi 127788, United Arab Emirates; c Department of Chemical Sciences, Parul Institute of Applied Sciences, Faculty of Applied Sciences, Parul University, Waghodia, Vadodara, Gujarat 391760, India

**Keywords:** Cavitation erosion-corrosion, Ultrasonic-assisted coatings, Acoustic cavitation, Corrosion science and Sonochemical Technologies

## Abstract

Corrosion causes substantial economic, safety, and environmental losses globally, motivating the development of advanced mitigation strategies. Recently, sonochemical technologies have emerged as versatile and powerful tools for actively controlling corrosion processes and understanding corrosion mechanisms. This review critically evaluates sonochemical principles and their role in corrosion and engineering. This review examines mechanisms of acoustic cavitation and their effects on metallic corrosion. Particular emphasis is placed on the interplay between sonophysical effects (microjet impregnation, shock waves, surface deformation, acoustic steaming) and sonochemical effects governing electrochemistry. The effect of ultrasound (US) parameters, such as power, reactor desing, exposure time, and frequency, is systematically presented in relation to diffusion-layer modulation, electrochemical performance, cavitation intensity, and coating densification. Beyond traditional effects, US is highlighted as a versatile tool that enhances interfacial adhesion, promotes surface activation, refines grain boundaries, and encourages uniform passivation-repassivation cycles, ultimately resulting in superior corrosion resistance. This review covers US-assisted corrosion protection strategies, with particular emphasis on efficient nanoparticle deagglomeration, intensified electrodeposition, enhanced corrosion-inhibitor adsorption, and improved coating integrity. Emerging US-enabled strategies, such as encapsulated corrosion inhibitors, US-grafted polymers, and self-healing coatings, are critically assessed. US is presented as a transformative technology in corrosion engineering, enabling controlled manipulation of surface phenomena despite potential cavitation damage. The work discusses potential pathways for industrial implementation, offers insights into emerging high-performance corrosion-mitigation strategies, and explores their potential for more resource-efficient processing. Despite significant advances, the competing effects of cavitation-induced degradation and corrosion mitigation remain poorly understood across material systems and operating conditions.

## Graphical abstract

**Figure:**
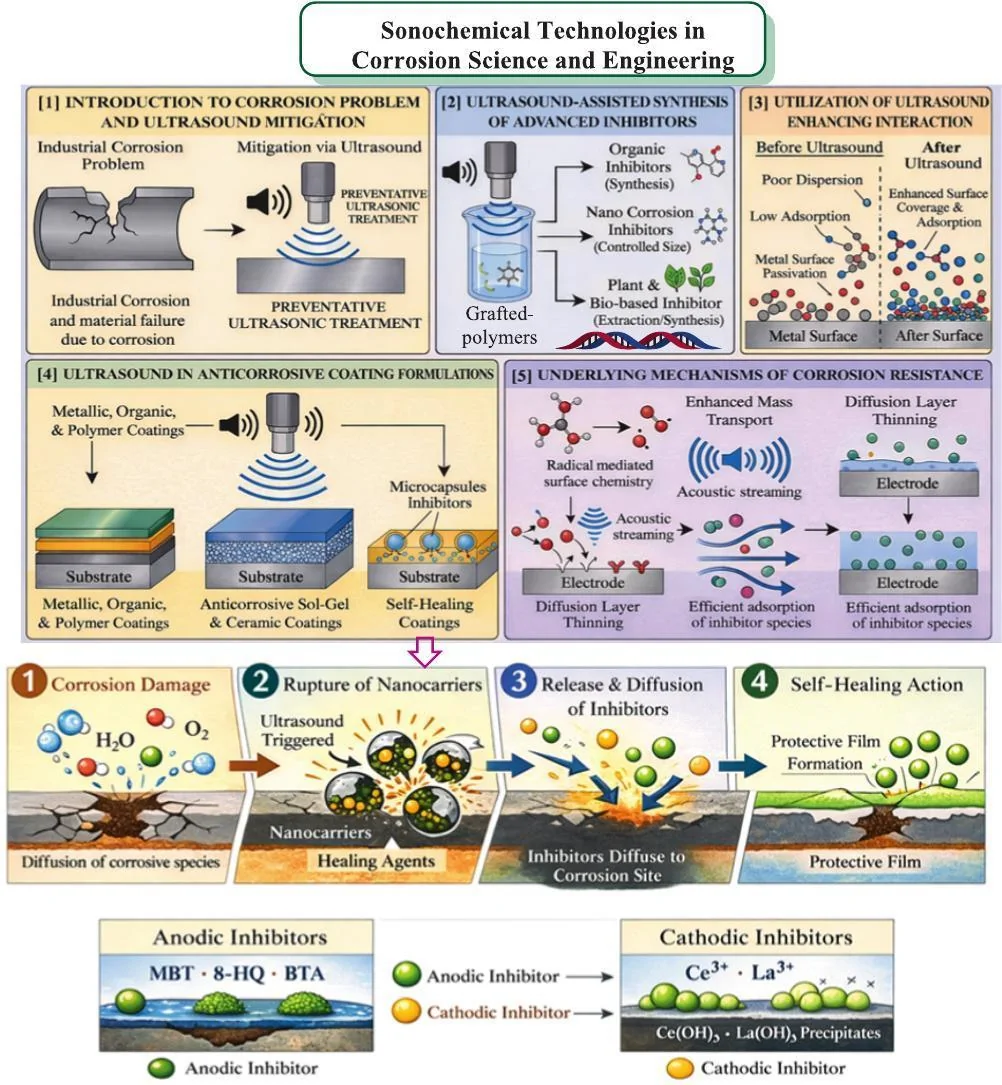

## 1 Introduction

### 1.1 Corrosion and corrosion mitigation: fundamental to advanced strategies

Corrosion has been widely studied, with global costs exceeding $2.5 trillion annually, equivalent to approximately 3–4 % of global GDP. Corrosion is a widespread problem that produces enormous safety, economic, and environmental risks [1]. Recent efforts have therefore focused on advanced corrosion protection strategies that integrate materials design and process intensification, including the use of corrosion inhibitors and surface coatings, which are tailored to the nature of the metallic substrates and environments [2]. Cathodic protection, corrosion inhibitors, and coatings remain widely used but are inadequate due to their passive operation and lack of dynamic interfacial control [3], [4], [5]. Recently, advanced corrosion mitigation strategies that combine innovative materials and smart science, engineering, and technologies to design protection systems have gained substantial attention [6], [7]. The use of nanotechnology-based coatings is one of these strategies, which involves the incorporation of nanoparticles such as graphene (G), graphene oxide (GO), carbon nanotubes (CNTs), nano-clay, or nano-silica in coating matrices to improve their strength, barrier properties, and resistance to the ingress of corrosive species [8], [9].

Recent advances in corrosion protection include nanocomposite coatings, self-healing systems, and smart corrosion inhibitors [10], [11]. Among these approaches, US has emerged as an adaptable process-intensification tool capable of improving mass transport, nanoparticle dispersion, coating formation, and surface activation. Nevertheless, its effect is inherently dualistic: modest cavitation improves corrosion resistance, whereas excessive cavitation may damage protective coatings and passive films. This review critically assesses these competing effects, summarizes recent developments, and discusses challenges associated with industrial implementation.x

### 1.2 Role of sonochemistry in corrosion science and engineering

**Fig. 1:**
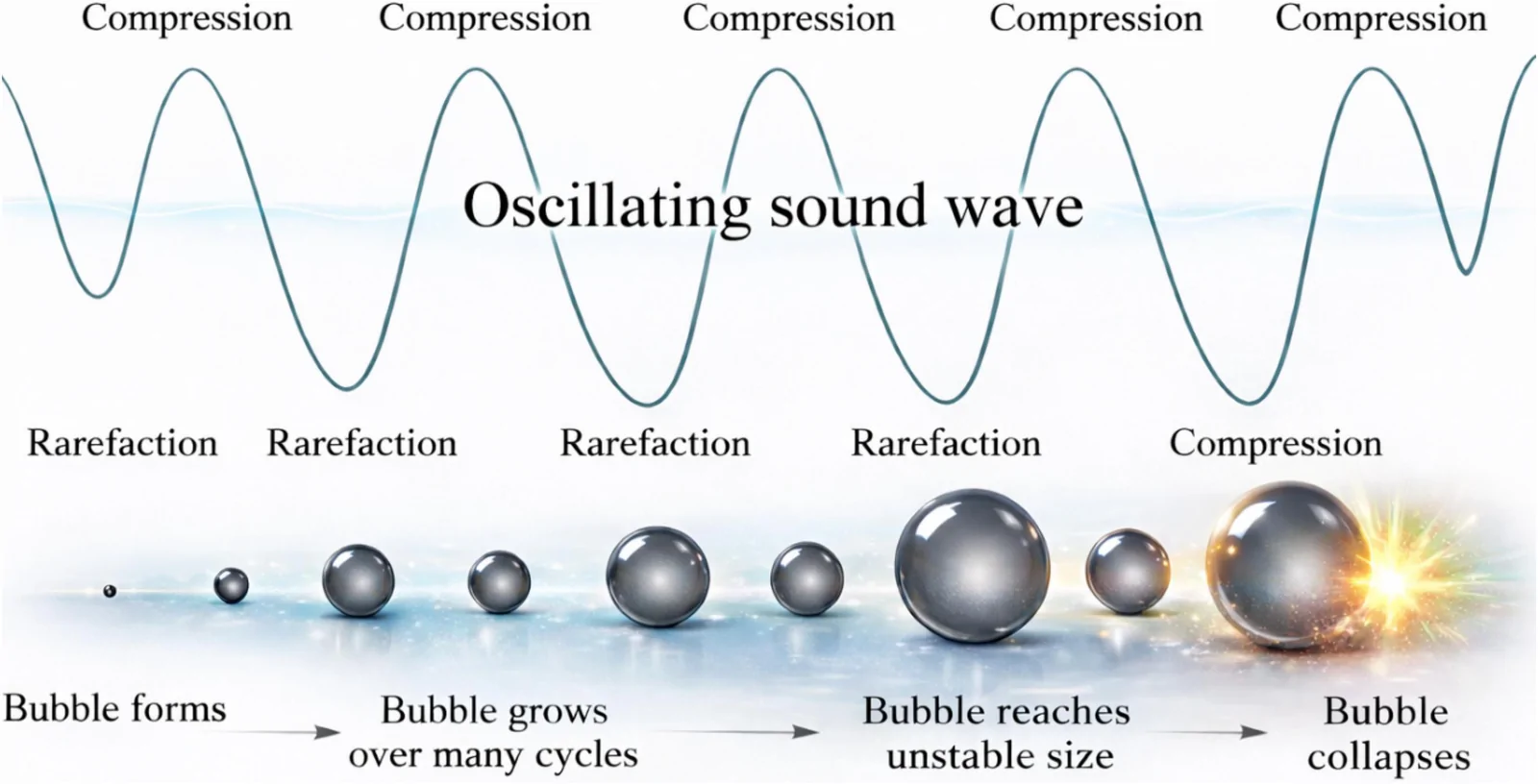
Schematic illustration of the process of bubble development, expansion, and collapse over several acoustic cycles.

Sonochemistry is the study of the chemical and physical effects induced by high-intensity US in liquids [12], [13]. Sonochemistry involves the formation of highly localized conditions via acoustic cavitation, thereby altering interfacial electrochemical processes. Considering corrosion systems, these alterations result in mechanical surface modification, radical formation, and increased mass transport, leading to both corrosion-mitigating and accelerating effects depending on the operating conditions. Cavitation involves bubble nucleation, growth, and collapse under alternating pressure fields, producing extreme localized conditions that affect electrochemical processes and surface morphology [14], [15]. Fig. 1 illustrates the cavitation process. Cavitation bubble collapse produces highly localized extreme conditions, with temperatures generally in the range of ∼4000–6000 K and pressures of several hundred MPa [13], [16], [17]. It is important to emphasize that these extreme pressures and temperatures represent estimated transient conditions inside collapsing cavitation bubbles during bulk collapse and do not represent the bulk liquid pressure or temperature. The collapse of the cavitation bubble near the solid surface (i.e., boundary collapse) becomes asymptotic due to the presence of the boundary, generating localized shock waves and high velocity microjets rather than a completely spherical implosion. Although local temperatures during boundary collapse may not be particularly high, the resulting mechanical effects, such as surface deformation, localized pressure pulses, and microjet impregnation, are often the primary contributors to erosion, corrosion, and coating modification. Moreover, cavitation intensity also greatly depends on liquid properties, including vapor pressure, temperature, viscosity, dissolved gas concentration, and surface tension. Dissolved gases play significant roles, serving as cavitation nuclei. They also modify collapse intensity and bubble stability, thereby influencing acoustic steaming, radical generation, and the balance between detrimental cavitation damage and beneficial surface activation. Therefore, the reported cavitation pressures and temperatures should be considered indicative values, with actual magnitudes depending on operating conditions and reactor configuration. This effect can adversely affect (damage) the metal surface and break the protective passive films. The intense local conditions caused by the US may have an adverse effect through damage, or a positive impact through improved surface properties that enhance corrosion resistance. Collectively, these effects and properties suggest that US does not produce a purely detrimental or beneficial effect; the balance between surface-stabilization and surface-damage mechanisms controls corrosion behavior in US-mediated systems.

US affects corrosion by three interconnected mechanisms: enhanced mass transport, sonophysical effects (microjets and shock waves), and sonochemical reactions involving radical formation. Their relative standing differs on the electrolyte, material, and ultrasonic operating conditions. Among the different forms of US-assisted degradation of materials, cavitation erosion-corrosion (CEC) is among the most common [18], [19], [20]. CEC frequently occurs in marine propellers, hydraulic turbines, fuel injectors, pumps, molten-metal processing systems, and US cleaning equipment. CEC occurs by a combined action of mechanical damage (erosion) and electrochemical dissolution (corrosion). CEC originated from the combined action of electrochemical dissolution and mechanical damage [21]. Cavitation improves ionic transport and minimizes concentration polarization by removing gas bubbles from the metallic surfaces [22], [23]. These processes enhance the density and uniformity of electrodeposited coatings. Costa and Neto reported improved polarization resistance at moderate US power; however, similar studies indicate deterioration in performance at higher intensities [24]. They observed and proposed that a controlled US power (200 W) greatly improves polarization resistance (R_p_) and reduces corrosion current density (i_corr_). Generally, refined surfaces are associated with a uniform passive film and a reduced propensity toward localized corrosion. In addition to these, US radiation can be used for the practical, green synthesis of corrosion inhibitors, particularly plant- and bio-based ones. Controlled sonicating can also accelerate the extraction of natural and synthetic corrosion inhibitors. The US technologies can also be used for corrosion detection. For example, nonlinear ultrasonics is useful for the early detection of corrosion-related microstructural changes, and high-resolution US imaging enables in situ visualization of localized corrosion (pitting or crevice) beneath corrosion products. US parameters such as power, frequency, reactor desing, and irradiation times control cavitation intensity and, therefore, coating effectiveness, passivation, and corrosion resistance. Their influences are discussed in detail in Section 3 [25], [26], [27], [28].

### 1.3 Corrosion-based sonochemical principles

**Fig. 2:**
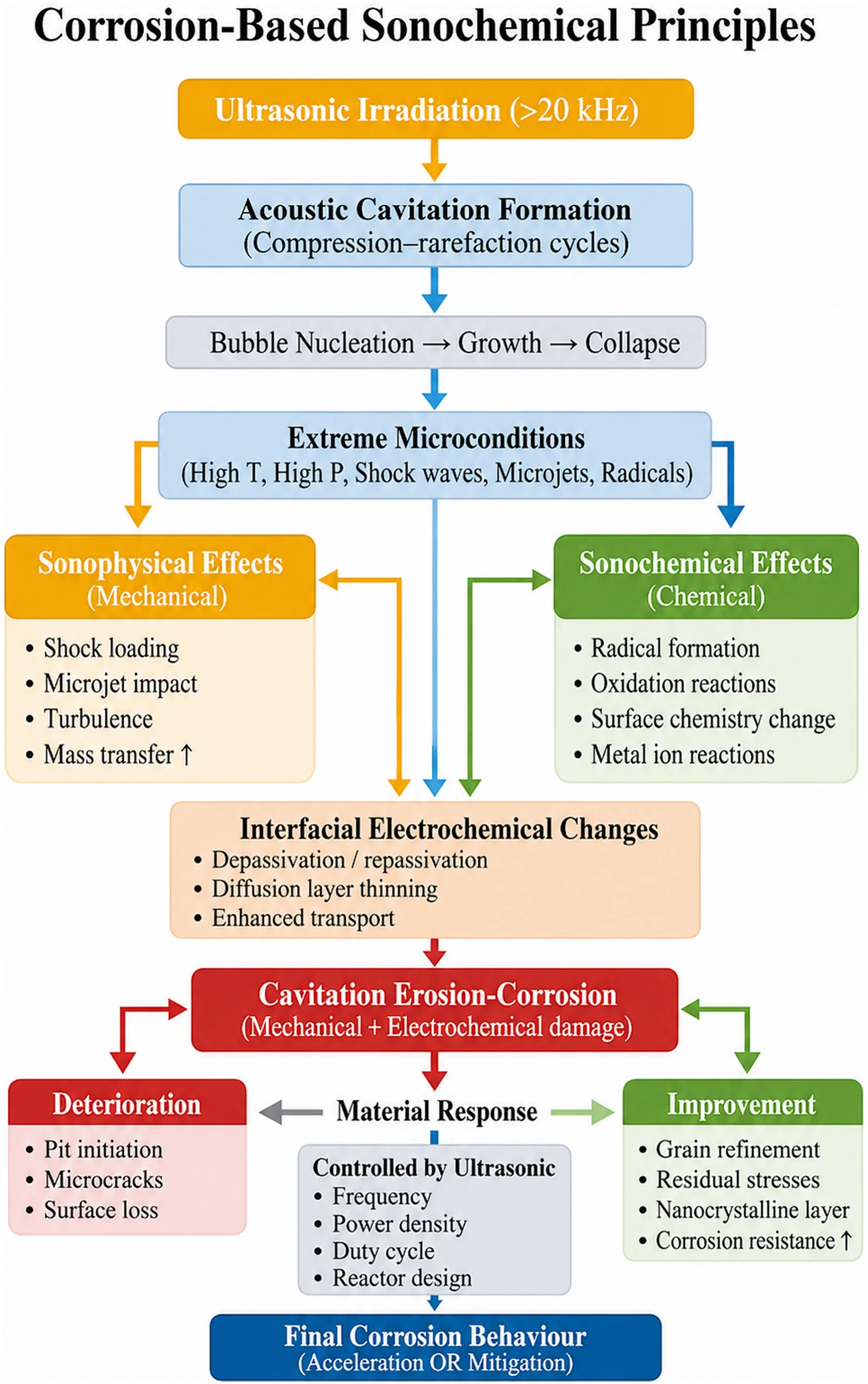
Flowchart illustrating the mechanism of corrosion-based sonochemical processes, showing the progression from acoustic cavitation to sonophysical and sonochemical effects, interfacial electrochemical changes, and resulting material response governed by US parameters.

Corrosion-based sonochemical processes arise from the interplay between electrochemical processes and cavitation dynamics, altering material behavior and producing localized extreme conditions [29], [30]. Corrosion behavior in US conditions is influenced by the interplay of electrochemical processes and cavitation dynamics, leading to both mechanical and chemical effects. Mechanical impacts arise from cavitation implosion, exemplified by shock pulse loading and micro-jet friction, while radical production in high-temperature bubble cavities accounts for chemical impacts. These combined effects significantly alter the corrosion mechanisms at solid-liquid interfaces, creating a dynamic depassivation-repassivation cycle essential for alloys that depend on durable oxide films for corrosion resistance [31]. Repetitive mechanical stress accelerates pit development and targeted corrosion in vulnerable materials like low-alloy steels. A key distinction in corrosion-related sonochemistry is between sonophysical and sonochemical effects. Sonophysical implications include mechanical and hydrodynamic outcomes of ultrasonic irradiation, such as acoustic transmission, boundary-layer diminution, turbulence amplification, shock-wave discharge, and micro-jet production [32]. These actions enhance mass-transfer kinetics by removing adsorbed coatings or corrosive substances from surfaces. Additionally, bubble rupture generates reactive species and microenvironments that drive chemical processes, thereby producing sonochemical effects. Reactive oxygen species, including hydroxyl radicals, can oxidize metal ions, alter surface chemistry, and degrade organic corrosion barriers. These processes occur in cycles and significantly impact various corrosion systems under US conditions (Fig. 2).

Cavitation erosion-corrosion results from a combination of mechanical damage and electrochemical breakdown induced by cavitation in corrosive electrolytes, often leading to greater material loss than either damage mechanism alone [21]. Corrosion weakens materials and accelerates fracture under stress, while cavitation creates slip zones and small cracks that become anodic sites [33]. In sonochemistry focused on corrosion, the material's response to ultrasound is influenced by operating conditions such as frequency, intensity, and reactor desing, which affect cavitation formation and consequently corrosion outcomes (Figure SI-1) [34].

US frequency significantly impacts bubble resonance size and disintegration velocity. Lower frequencies (20-40 kHz) enhance cavitation strength, leading to effective surface cleaning and higher mechanical stresses [35]. In contrast, higher frequencies typically promote the formation of smaller cavitation bubbles and may enhance radical generation under appropriate operating conditions. However, the comparative contributions of sonochemical and sonophysical effects depend not only on frequency but also on acoustic pressure, liquid properties, temperature, dissolved gas content, and reactor configuration [17], [26], [35]. Although ultrasonic frequency influences cavitation behavior, it should not be considered the sole determinant of sonophysical or sonochemical activity. The dominant mechanism depends on the combined effects of frequency, reactor desing, acoustic pressure, dissolved gas concentration, liquid properties, and operating conditions. Stable cavitation enhances mass transport and diffusion, thereby reducing the thickness of the diffusion layer and increasing the critical current density in electrochemical processes. At elevated power densities, transient cavitation causes significant surface pitting and shock waves. The extreme plastic distortion induced by methods such as US nanocrystal surface modification (UNSM) leads to grain optimization and increased dislocation density [36], [37]. In cavitation-corrosion scenarios, these changes help mitigate the initiation of fractures and pits. A thin nanocrystalline coating from US treatment boosts stiffness and resistance to cavitation fatigue. Additionally, restrictive residual stresses in cavitating environments reduce material loss and enhance durability while altering interfacial electrochemical rates [38].

**Fig. 3:**
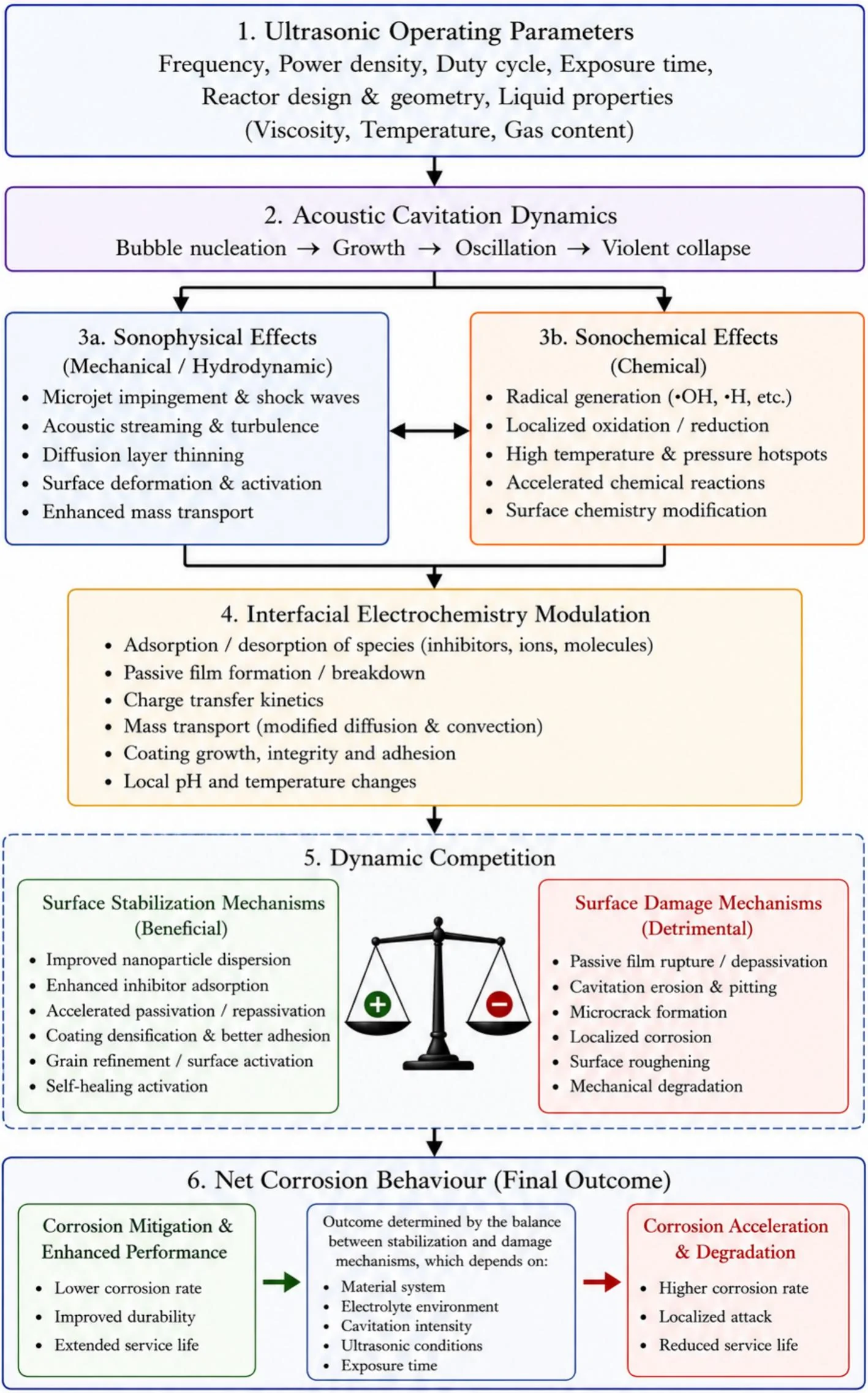
Schematic illustration of the proposed framework showing the interaction of US cavitation, interfacial electrochemistry, and corrosion behavior.

Despite advancements in corrosion science and sonochemistry, interactions between electrochemical corrosion and US cavitation have typically been viewed in isolation. This article proposes a unified framework in which corrosion behavior under US irradiation results from the interplay of interfacial electrochemistry, material response, and cavitation physics, influenced by factors such as acoustic power density and liquid properties (Fig. 3). Cavitation effects are categorized as sonophysical (e.g., microjet impregnation, acoustic steaming) and sonochemical (e.g., radical formation, enhanced chemical reactions), which modify the electrochemical interface by altering kinetics and transport processes. Consequently, corrosion responses arise from a dynamic competition between detrimental (e.g., surface erosion, film rupture) and beneficial effects (e.g., improved passivation, coating formation). The overall corrosion performance reflects this balance, varying with electrolyte chemistry and US conditions: moderate cavitation enhances stabilization, while excessive cavitation leads to degradation. This framework provides insights into the contrasting findings in the literature and helps optimize US processing conditions to reduce corrosion.

The literature included in the present review was identified through searches of the ScienceDirect, Google Scholar, Scopus, and Web of Science databases using keywords such as ultrasound, acoustic cavitation, sonochemistry, corrosion, passivation, corrosion inhibition, and protective coatings. This review primarily considered the publications from 2000 to 2025. Publications were designated based on their relevance to US-assisted corrosion science, electrochemical performance, mechanistic understanding, and engineering applications, whereas duplicate and unrelated publications were excluded.

## 2 US-assisted synthesis of corrosion inhibitors

### 2.1 US-assisted synthesis of organic corrosion inhibitors

US-based synthetic strategies, particularly US and microwave (MW) heating, have proven efficient for synthesizing corrosion inhibitors with improved reaction kinetics and reduced processing time [39], [40], [41], [42], [43], [44], [45], [46], [47], [48], [49]. Research highlights various corrosion inhibitors, including heterocycles, surfactants, and Schiff bases, synthesized through US-assisted techniques, achieving high inhibition efficiencies (>90%) (Table SI-1). These inhibitors generally function as mixed-type inhibitors, effectively hindering both anodic and cathodic reactions, with efficiencies ranging from 95% to 98%. The nature of substituents significantly influences their inhibition potential, with electron-donating groups enhancing adsorption. However, excessive cavitation during synthesis may degrade sensitive compounds, necessitating careful optimization of process conditions such as frequency, power, temperature, and sonication time to minimize degradation and maximize benefits. The literature survey shows that although ultrasonic synthesis generally shortens reaction times, claims of environmental sustainability or energy efficiency should be treated with caution, as quantitative data on electrical energy consumption, solvent use, waste generation, and life-cycle performance are rarely reported.

### 2.2 US-assisted synthesis of green (plant- & bio-based) corrosion inhibitors

The use of natural materials is progressively explored as a potentially more environmentally compatible approach [[50], [51], [52], [53], [54]]; however, the overall sustainability of US-assisted extraction depends on factors such as solvent use, extraction yield, energy consumption, and waste generation. The effectiveness of these extracts in preventing corrosion is influenced by factors such as extraction methods, molecular weight, and phytochemical nature. Two notable methods are ultrasound-assisted extraction (UAE) and ultrasound-assisted enzymatic extraction (UEE), which are preferred over traditional extraction techniques due to their efficiency [55], [56], [57], [58]. Ultrasound is employed in three primary ways: extracting corrosion inhibitors from biomass, modifying biomolecules for improved functionality, and purifying fractions. Recent studies demonstrate that UAE significantly enhances the extraction of phytochemicals, yielding inhibition efficiencies (%IE) ranging from 85 to 96% across various substrates. Effective plant extracts, such as okra pectin, exhibit mixed-type inhibition, impacting both anodic and cathodic reactions by forming barriers at metal-electrolyte interfaces. Comparative studies highlight the superior effectiveness of UAE-derived extracts versus traditional methods, emphasizing the role of phytochemical properties in corrosion inhibition. Table SI-2 summarises major reports on UEE and UEE plant extract-based corrosion inhibitors [[59], [60], [61], [62], [63], [64], [65], [66]].

### 2.3 US-assisted grafted polymeric corrosion inhibitors

Recently, the use of grafted polymers in corrosion protection has gained significant attention [67], [68]. Grafting offers several advantages, including tailoring their properties and increasing the density of functional (adsorption) groups. Several adsorption sites, such as polar functional groups, aromatic rings, π-conjugated systems, and heteroatoms (S, O, N, P), can be incorporated into polymer molecular structures to enhance their bonding to metal surfaces. Generally, grafting provides a branched architecture and molecular size that enhance surface coverage and protection, accelerate the formation of stable, compact protective films, and minimize active corrosion sites [69], [70], [71]. Moreover, grafting can increase the dispersion of corrosion inhibitors, enhance their solubility, and improve their compatibility with corrosive electrolytes [69], [70], [71] . These properties essentially overcome the poor crystallinity and solubility limitations associated with traditional polymeric corrosion inhibitors. The use of US-assisted grafted polymers could be an effective alternative for grafting, given their cost, time, and eco-friendliness [63], [72], [73].

The US-assisted grafting of polymers provides efficient, homogeneous grafting without prolonged heating. US cavitation generates enormous, localized heat and pressure, accelerating radical formation and subsequent grafting. Dalhatu et al. developed US-assisted chitosan grafted with poly(2-hydroxyethyl methacrylate) (CS-g-PHEMA) and evaluated its inhibition efficiency against mild steel in 0.2 M H_2_SO_4_ [74]. FT-IR and thermal analyses were used to confirm the grafting process. These analyses revealed that PHEMA was successfully and effectively incorporated into the chitosan backbone. Weight-loss and electrochemical studies showed that CS-g-PHEMA significantly reduced the corrosion rate (C_R_) and achieved nearly 90% efficiency at 500 ppm. The results also showed that CS-g-PHEMA increases the barrier for the corrosion process by increasing the activation energy (E_a_). Potentiodynamic polarization study revealed that CS-g-PHEMA effectively inhibits both anodic metal corrosion and cathodic hydrogen evolution reactions, without significantly shifting the corrosion potential (E_corr_), indicating it is a mixed-type corrosion inhibitor. The literature study reveals that reports on the corrosion-inhibition potential of grafted polymers are scarce, leaving significant gaps for future studies, especially for comparing the corrosion-inhibition potential of traditionally grafted polymers with that of US-assisted grafted polymers.

The critical literature analysis demonstrates that although US-assisted corrosion inhibitor synthesis is associated with reduced reaction times and inhibition efficiencies exceeding 90%, direct comparison across the reported studies remains highly challenging due to considerable variation in experimental conditions. Immersion time, inhibitor concentration, electrolyte composition, electrochemical testing protocols, and the nature of the metallic substrate differ substantially, making quantitative comparison of inhibition performance potentially misleading. Beyond these, most existing studies remain limited to short-term laboratory-based testing, especially in acidic electrolytes. In contrast, long-term stability, adsorption mechanisms, bonding nature, durability, and industrial uses remain largely unexplored. Therefore, future studies should emphasize standardized electrochemical testing protocols and measure and report corrosion inhibition performance under more realistic conditions, especially in industrial environments. To facilitate quantitative comparison among the articles considered, the summary tables in this manuscript report US operating conditions and corrosion testing parameters, where available. Moreover, several publications do not consistently report key experimental parameters, including acoustic power density, treated volume, probe geometry, colorimetric power, duty cycle, dissolved gas conditions, solution temperature, and probe-to-specimen distance. The need for standardized reporting to improve reproducibility and enable meaningful comparison across studies has been highlighted.

## 3 US in improving corrosion inhibitors performance

### 3.1 US in enhancing nanoparticle dispersion and deagglomeration

Advancements in corrosion protection are enhanced by nanoparticles, which improve barrier performance and mechanical strength in coatings. Common nanoparticles include SiO_2_, TiO_2_, ZnO, Al_2_O_3_, carbon nanotubes, graphene oxide, and metallic particles, integrated into various coating systems. However, limitations arise from nanoparticles' tendency to clump due to van der Waals forces and electrostatic interactions, which can cause defects that compromise durability. US irradiation is noted as an effective technique for improving nanoparticle deagglomeration through localized mechanical forces [75]. Acoustic cavitation is the phenomenon in which ultrasound waves create bubbles in liquids that collapse, producing high-velocity microjets and shock waves, which are particularly effective near nanoparticle clusters. This results in strong shear stresses that break larger agglomerates into smaller, stable pieces, reducing particle attraction. Moreover, acoustic steaming promotes better particle distribution in the suspension [26], [76]. However, high ultrasound intensity can cause interfacial defects and nanoparticle fragmentation, thereby negatively impacting long-term performance. The distribution of nanoparticles significantly affects a coating's effectiveness in preventing corrosion. A uniform distribution creates a maze-like structure that slows the passage of harmful substances, such as oxygen and chloride ions, thereby enhancing barrier performance and reducing permeability [77], [78]. Conversely, poor dispersion results in particle clustering, leading to structural defects that increase porosity and allow corrosive agents to accumulate, thus promoting localized corrosion through microgalvanic effects or weak bonding zones [79], [80].

Introducing nanoparticles into polymer-based coatings such as epoxy and acrylic enhances mechanical performance and corrosion resistance. The US processing technique addresses particle clumping, ensuring a uniform distribution within the polymer matrix, especially before curing. This leads to a more stable cellular structure, improving the coating's electrochemical behavior, corrosion resistance, and impedance. CNTs and GO are prone to agglomeration due to strong interactions between them. When well distributed, GO reduces chloride-ion permeability in epoxy, thereby enhancing corrosion protection [81]. Conversely, agglomerated graphene can create a network that accelerates galvanic corrosion. US sonication effectively separates CNT bundles and peels graphene oxide layers, enhancing their reinforcement performance [81]. The US plays a crucial role in metallic composite coatings such as Ni-SiC and Ni-graphene by using ultrasonic agitation to enhance nanoparticle incorporation during electrodeposition, thereby improving coating compactness. Controlled cavitation in plating baths, where hydrogen bubbles detach from the cathode, creates denser deposits with fewer defects [82]. The relationship between ultrasonic power and dispersion efficiency is nonlinear: excessive power can damage nanoparticles, while insufficient power fails to generate the necessary shear forces, leading to agglomeration and poor coating performance. Properly adjusted ultrasonic strength regulates cavitation, resulting in a more homogeneous microstructure, reduced porosity, and increased corrosion resistance.

### 3.2 US in enhancing surface coverage and adsorption/adhesion

Surface coverage, adsorption, and interfacial adhesion are vital to the performance of corrosion-protective systems such as coatings and inhibitors. The success of corrosion protection relies on the material properties and the strength of the bond with the substrate. Incomplete coverage can result in localized corrosion and premature failure. US treatment enhances surface coverage, adsorption, and adhesion by inducing acoustic cavitation and other effects that improve surface cleaning and bonding, thereby providing better corrosion protection [53], [54], [55], [56]. The primary mechanism for significant surface modification by US is acoustic cavitation, in which high-intensity US waves induce compression and rarefaction cycles in a liquid, leading to bubble formation and collapse. The bubble implosion generates extreme localized conditions such as high temperatures and pressures, producing shear stresses that can remove oxide layers and impurities, increasing microscale surface roughness. Furthermore, acoustic steaming aids mass transport, reduces diffusion boundary layer thickness, and enhances the flux of reactive species at the solid-liquid interface, speeding up adsorption kinetics and promoting uniform surface coverage [83], [84].

In corrosion inhibition systems, effective surface coverage is critical as organic inhibitors protect metallic surfaces by forming a barrier that reduces anodic and cathodic reactions. In static systems, adsorption can be hindered by slow diffusion and surface heterogeneity. However, US irradiation enhances adsorption through mechanisms such as increased mass transfer, surface activation that removes oxide layers, and improved molecular packing, leading to stronger interactions. US-assisted systems exhibit higher adsorption densities and stronger film adhesion, resulting in improved inhibition efficiency (Figure SI-2). Electrochemical measurements indicate reduced corrosion current densities, higher polarization resistance, and larger Nyquist semi-circles, confirming enhanced surface coverage and stability. Adsorption behavior is analyzed using Langmuir or Temkin isotherm models, with US conditions yielding increased adsorption rate constants and improved interfacial mass transport.

**Fig. 4:**
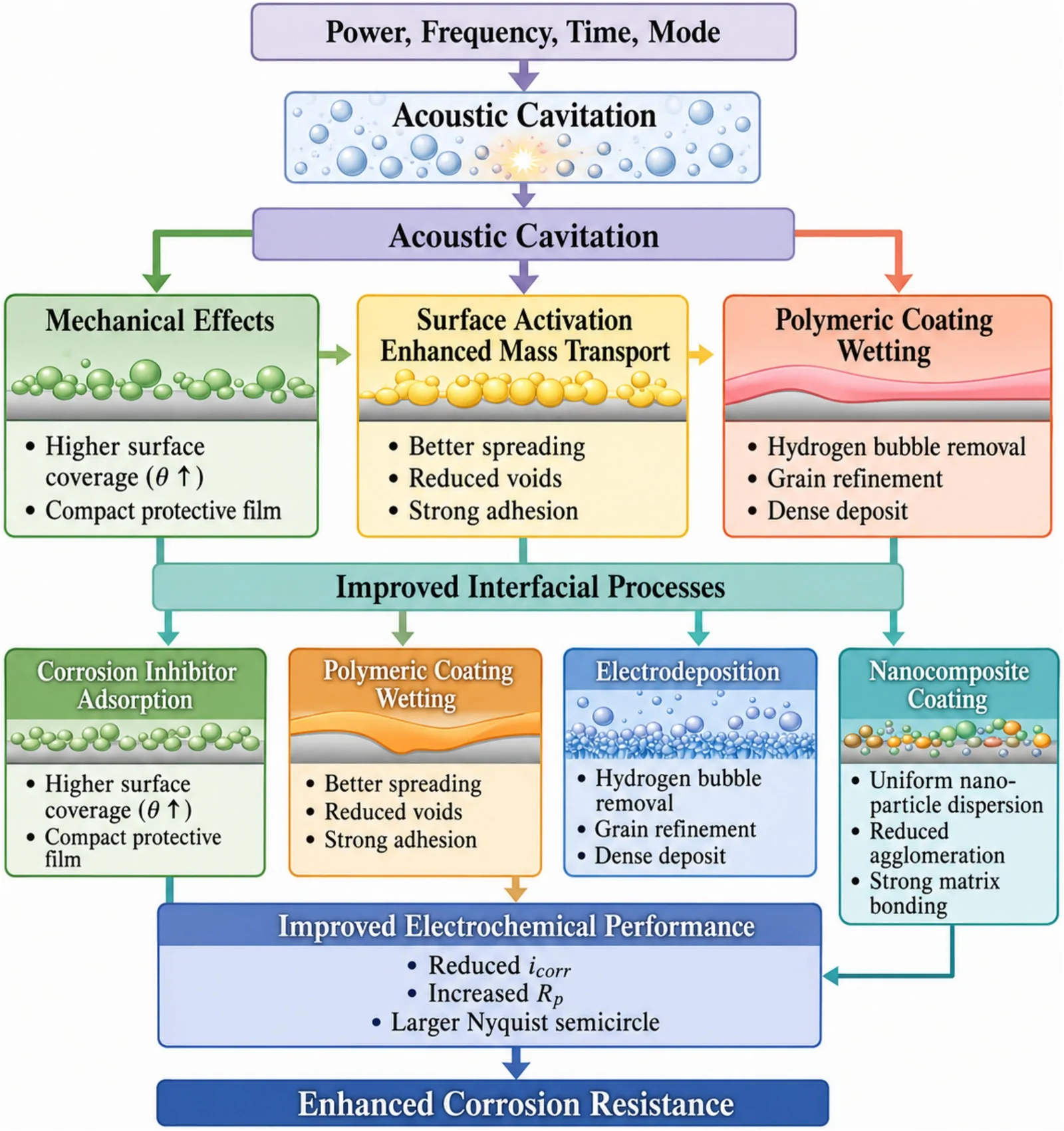
Schematic illustration of the US-driven pathway for enhanced surface coverage as well as corrosion protection. US cavitation promotes surface activation and enhanced mass transport, leading to better adsorption, coating quality, electrode positioning, nanoparticle dispersion, and, ultimately, improved electrochemical performance and corrosion resistance.

For polymeric corrosion-protective coatings, complete wetting and uniform spreading are vital for barrier integrity, as poor wetting can cause defects that reduce corrosion resistance. Ultrasonic treatment enhances wetting by lowering interfacial tension and enabling deeper penetration of the coating. This method also cleans surfaces by removing contaminants, increasing surface area for better mechanical interlocking, and exposing active sites for stronger chemical bonds, thus minimizing the risk of delamination. In electrodeposition, US enhances surface coverage by improving ion transport and removing hydrogen bubbles, which can create pores and defects that degrade coating quality. US cavitation dislodges these bubbles, resulting in smoother, denser deposits [61]. Additionally, acoustic steaming reduces the diffusion layer thickness and increases the limiting current density, thereby raising deposition rates, refining grain structure, enhancing coating uniformity, and minimizing defects. In composite electrodeposition, US agitation promotes better nanoparticle dispersion and bonding within the metallic matrix (Fig. 4).

Electrochemical Impedance Spectroscopy (EIS) under irradiation necessitates careful interpretation due to deviations from conventional measurement assumptions. Unlike standard EIS, which assumes a linear, stationary electrochemical system, ultrasound introduces variables such as acoustic streaming and changing diffusion boundary layers, resulting in transient responses rather than steady-state conditions. Consequently, observed changes in charge-transfer resistance (R_ct_), R_p_, and double-layer capacitance (C_dl_) are influenced by hydrodynamic effects and intrinsic kinetics rather than by enhanced kinetics alone. In particular, the reduction in the Warburg diffusion component is attributed to convective mass transport associated with thinner diffusion layers and acoustic steaming. For accurate comparisons between US and non-US EIS data, consistent ultrasound conditions are recommended, ideally during stabilized pulsed sonication or after transient cavitation effects stabilize.

The influence of US power on surface coverage and adhesion is nonlinear. Low power fails to modify surfaces, while moderate power optimizes cavitation for effective cleaning and enhanced adhesion. Excessive power risk erodes and reduces coating quality. Insufficient sonication may impede activation, whereas prolonged exposure can damage productive layers. Optimal sonication duration maximizes adsorption density and maintains structural integrity. In nanocomposite corrosion-protective systems, durable performance relies on robust interfacial bonding among nanoparticles, the matrix, the coating, and the substrate. Ultrasonication improves nanoparticle dispersion, limits agglomeration, and decreases interfacial voids, thereby reducing moisture ingress and inhibiting crack growth in corrosive conditions. Furthermore, sonochemical reactions during ultrasonication generate reactive radicals that enhance surface chemistry and functionalize nanoparticle surfaces, further reinforcing interfacial bonding and adhesion stability [54]. Reactor configuration is essential for enhancing surfaces in the US. Probe-type ultrasonicators provide high energy density for activation and sono-electrochemical applications, while bath-type systems offer uniform yet lower intensity treatments. Industrial corrosion protection requires multi-transducer setups in flow-through reactors for effective acoustic field distribution and surface modification [55]. Pulsed ultrasound minimizes thermal accumulation and stabilizes adsorption layers, enhancing mass transport and reducing the risk of surface erosion and film disruption [56].

### 3.3 US in enhancing the passivation of metallic surfaces

US treatment enhances passivation by facilitating mass transport, surface activation, and rapid depassivation-repassivation cycles, primarily through acoustic cavitation [54], [56]. High-intensity ultrasound (US) generates microbubbles that clean surfaces and disrupt unstable oxide layers, while acoustic steaming increases the transport of oxygen and metal ions [54], [56]. This results in the formation of compact and low-defect-density passive films (Figure SI-3) [53], [63]. The dynamic processes of film removal and growth allow for immediate repassivation upon exposure of fresh metal surfaces. Subsequent studies reveal that US modifies electrochemical parameters, decreasing the diffusion layer thickness and enhancing electrode surface renewal, thereby improving oxide film quality during anodizing.

US effectively enhances the growth of passive films, helping prevent localized failure often initiated at defects in these films. In chloride-related environments, US increases metal resistance to corrosion by facilitating rapid repassivation, resulting in higher potentials than in the absence of US. The relationship between US power and passivation is non-linear: low intensities do not significantly affect mass transport, while moderate levels lead to uniform, compact film growth. However, high intensities can erode passive films, increasing metal dissolution, indicating an optimal power range for effective passivation. Improved polarization resistance and reduced corrosion current in stainless steel result from ultrasonic treatment, which enhances chromium enrichment in the passive film. In aluminum alloys, ultrasonic-assisted anodizing leads to thicker anodic oxide films with better barrier properties, accelerates oxide growth, and enhances titanium oxide adhesion [53], [62]. Optimal exposure time is crucial to avoid surface damage or inadequate protection. Ultrasonic treatment also enhances chemical passivation, surface coverage, and coating adhesion. Reactor desing should optimize ultrasonic distribution, with probe-type devices for precise energy focus and bath-style methods for uniform energy. For industrial applications, multiple US transducer configurations or flow-through reactors effectively apply cavitation energy. A pulsed ultrasonic method stabilizes passive films, improves mass transport, and minimizes mechanical disruption, enhancing film integrity and stability. The temporal evolution of passive film quality under US treatment is shown in Figure SI-4.

Cavitation removes contaminants and unstable oxide fragments, thereby enhancing corrosion resistance by promoting rapid oxidation and the formation of denser oxide films. US aids sonochemical reactions and radical generation, thereby promoting oxidation and passive film formation on metals. While excessive cavitation can damage the film, optimizing conditions is essential. Efficiency hinges on sound-field distribution and reactor desing, with specific systems tailored to various applications. US treatment enhances metal surface passivation via microjets for cleaning and activation. At the same time, acoustic steaming reduces the diffusion layer thickness, thereby accelerating electrochemical reactions and producing a more uniform passive layer. Optimally, the passive film is denser with fewer defects, leading to improved localized corrosion resistance and durability. US-assisted passivation is increasingly viewed as a sustainable method for extending metal service life in harsh environments.

### 3.4 US in encapsulated inhibitors and controlled release systems

Recent advancements in environmentally sustainable smart corrosion protection systems utilize encapsulated corrosion inhibitors with controlled-release technologies. Traditional inhibitors often face limitations due to rapid consumption and environmental concerns. New encapsulation methods involve microcapsules, nanocontainers, and other materials that release inhibitors in response to environmental triggers like pH changes or mechanical damage. US has emerged as an effective method for improving encapsulation and release [85]. It enhances encapsulation efficiency, controls particle size, and facilitates inhibitor release via acoustic cavitation [17]. These systems enhance the self-healing capacity of coatings by activating inhibitors when aggressive ions, such as chlorides, penetrate damaged areas. The success of these systems relies on uniform distribution, microcapsule adhesion, and controlled release kinetics, all of which are improved by US techniques [86]. The encapsulation mechanism under US treatment is illustrated in Figure SI-5.

**Fig. 5:**
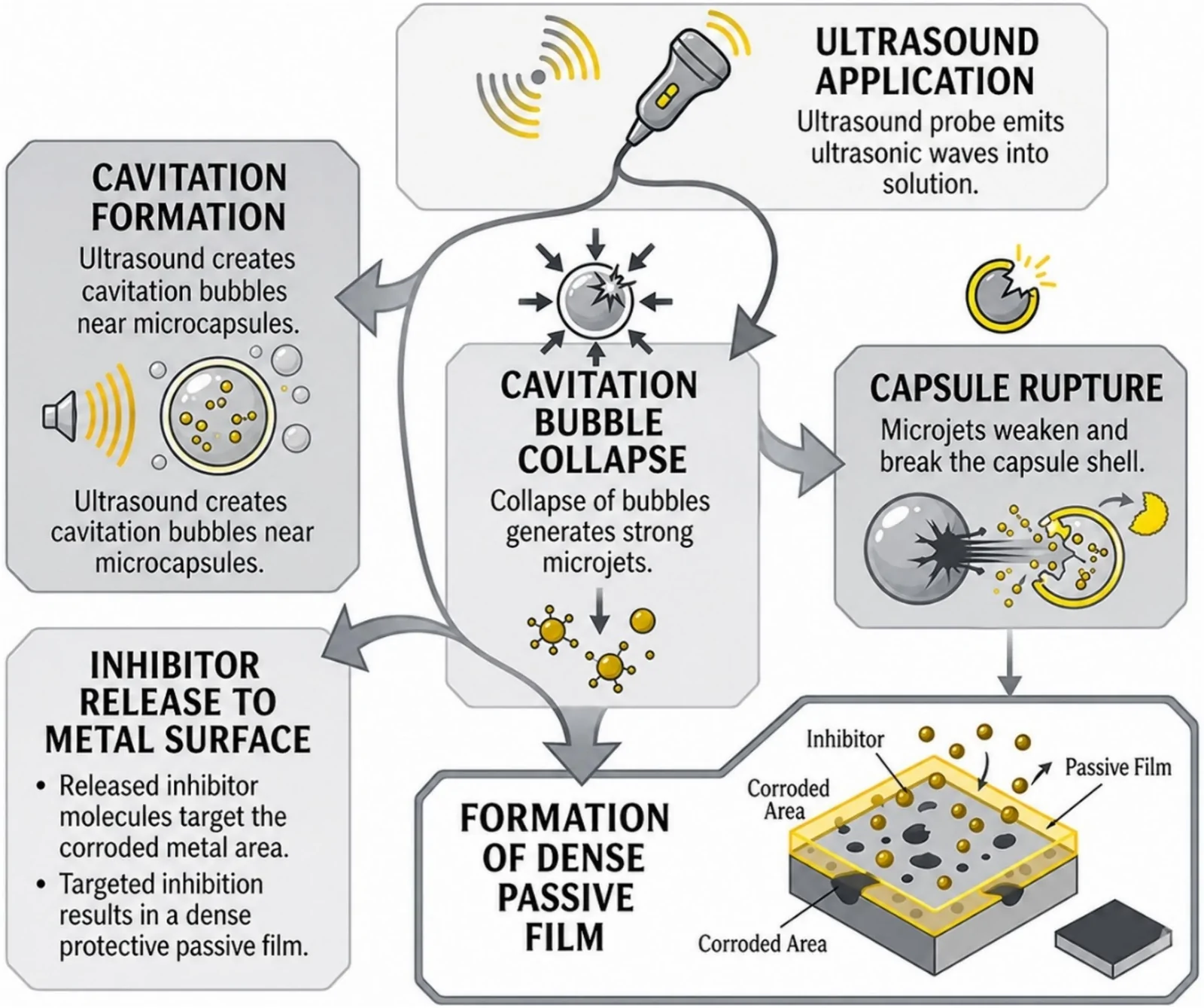
US-triggered release of corrosion inhibitors. Cavitation bubble collapse near encapsulated particles generates microjets that weaken or rupture the capsule shell, allowing controlled release of inhibitor molecules toward the damaged metal surface.

In addition to microcapsules, mesoporous silica nanoparticles (MSNs), halloysite nanotubes, and layered double hydroxides (LDHs) serve as nanocontainer systems for inhibitor storage and release. US enhances the loading efficiency of inhibitors by improving diffusion and accelerating mass transport to nanopores, resulting in higher encapsulation efficiency than static conditions [87]. In LDH systems, US enhances ion exchange, facilitating anion diffusion into interlayer spaces. Effective release kinetics are crucial for the long-term protection of encapsulated inhibitors; rapid release can deplete inhibitors prematurely, while slow release may allow damage to occur. US influences release kinetics via passive structural modifications or active triggering of release mechanisms. Specifically, localized US exposure can rupture capsules or increase inhibitor diffusion through microjet cavitation. This allows for the development of externally controllable self-healing coatings. The release mechanism under US activation is shown schematically in Fig. 5.

The release kinetics of US-induced corrosion inhibitors depend on capsule shell thickness, composition, and the applied acoustic energy density. At lower energy densities, cavitation activity is minimal, leading to slow inhibitor release primarily through diffusion, which is critical for sustained corrosion mitigation. In contrast, higher energy densities enhance cavitation, causing shell ruptures or permeabilization that increase release rates but may also lead to rapid depletion and degradation of inhibitors. An optimal energy density range balances controlled release and prolonged protection. Moreover, US enhances the distribution of encapsulated materials within coatings, preventing agglomeration and voids, thus improving mechanical integrity [86], [88]. Coatings produced via US-assisted methods demonstrate superior corrosion resistance, as reflected in higher electrochemical impedance than coatings produced by mechanically mixed methods, a difference attributed to improved capsule dispersion and adhesion. The application of US enhances the adhesion of encapsulated particles to coatings by introducing roughness and functionalization to the capsule shell, improving mechanical interlocking and chemical compatibility. This leads to reduced detachment and increased durability. The relationship between US power, encapsulation, and release is nonlinear: low power fails to produce uniform capsules, while excessive power compromises capsule integrity and may damage inhibitor molecules. Optimal mid-range power is necessary for effective shell formation. Moreover, the duration of US application affects encapsulation quality; short durations may lead to incomplete shells, while longer durations could harm capsules and diminish inhibitor efficacy. In self-repairing coatings, US improves dispersion and response by standardizing capsule size, enhancing the release mechanisms sensitive to pH or chloride. Figure SI-6 illustrates the influence of US on inhibitor-release behavior over time.

The inhibitor molecules are released from capsules in response to environmental or US stimuli, providing sustained surface protection. Reactor desing is crucial for industrial applications: probe-type ultrasonicators are suitable for lab-scale processes, whereas flow-through reactors are needed for uniform droplet distribution at larger scales. Maintaining constant volumetric power density is essential for reproducibility during scale-up. US pulses enhance encapsulation by stabilizing capsules, reducing damage, and promoting sonochemical reactions that strengthen shells and control release [17]. Effective parameter optimization is necessary to avoid inhibitor degradation and excessive energy use, while US irradiation improves encapsulation efficiency and corrosion resistance. Future studies should aim for encapsulation processes that translate easily to industrial production, emphasizing uniformity and efficiency. Current research often focuses on a single optimization parameter, highlighting the need for a more comprehensive approach to reactor desing.

## 4 Ultrasound in anticorrosive coatings

### 4.1 US in anticorrosive metallic coatings

**Fig. 6:**
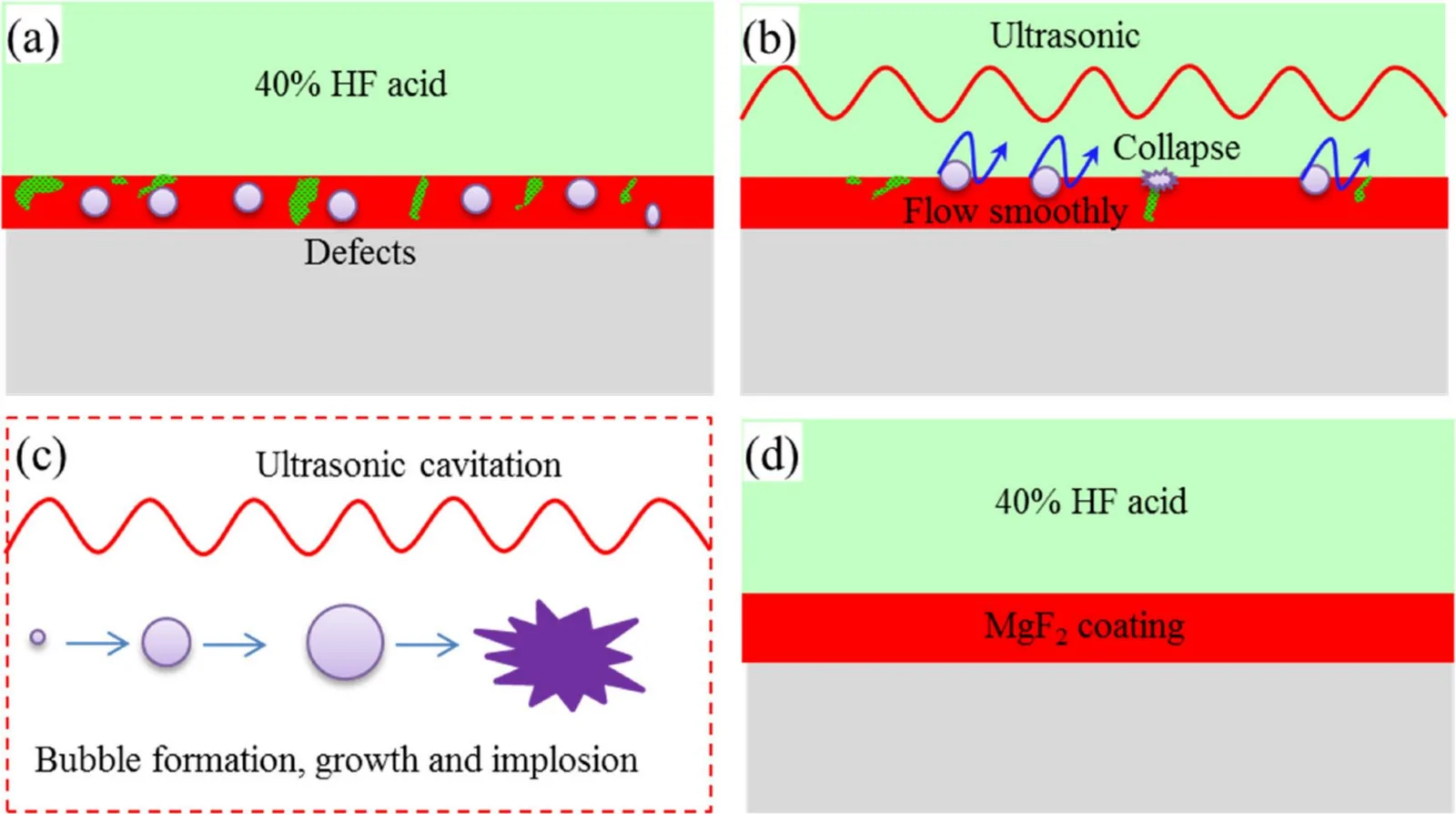
Diagrammatic representation of the protective properties of F- and U-treated magnesium alloys: (a) the F-treated magnesium alloy, (b) the U-treated magnesium alloy, (c) the effects of US cavitation during liquid US treatment, and (d) the ultrasonically treated fluoride coating free of cracks or pore defects [96]. (Reproduced from an open-access journal, © 2020, No permission required).

Anticorrosive coatings protect metallic structures through three primary mechanisms: physical barrier protection, sacrificial anode protection, and passivating protection (Table SI-3). Physical barrier protection prevents corrosion by isolating metals from harmful environments. Sacrificial anodic protection involves a more active metal serving as an anode to protect a noble counterpart through galvanic coupling [89], [90], [91]. Passivating protection incorporates corrosion inhibitors like Ce-based coatings, which enhance cathodic protection by forming insoluble products [92]. The effectiveness of these coatings depends on their surface properties and stability, and they outperform traditional organic coatings in thermal stability and adhesion [93], [94], [95]. Recent studies highlight the benefits of US treatment in enhancing the quality of metallic coatings by improving density, surface smoothness, and corrosion resistance, demonstrated in experiments with MgF_2_ coatings on AZ31 alloys. US treatment reduces surface defects and enhances the overall protective capabilities of anticorrosive coatings. Li et al., while studying US-mediated MgF_2_ coatings on AZ31 Mg alloy, observed that the metallic coating reduced the *C*_R_ and decreased *i*_corr_ [96]. They observed that in the absence of US treatment, coated structures developed numerous surface cracks and pores due to H_2_ evolution via reactions between Mg and HF. However, the US-treated surface had fewer surface defects. After US treatment at 28 kHz, the surface becomes smoother, denser, firmer, and free of visual surface defects. The US treatment smooths the surface via cavitation Fig. 6.

The literature review discusses the anticorrosive performance of Ni/GO and Ni-W alloy-based composite coatings [24], [97], [98]. Wang et al. evaluated CaCO3/Mg(Al) bilayer coatings on AZ41 Mg alloys in chloride solutions, finding that US treatment enhances the density of the CaCO_3_ layer and the passivation of the inner Mg-Al-O layer, leading to lower *C*_R_ and i_corr_ values [99]. The Nyquist plots showed the largest capacitive loop and the highest *R*_ct_ in the Nyquist plots after 96 hours. Notably, the impedance modulus increased over time, indicating repassivation. Additionally, US treatment improved the hardness, compactness, and durability of the coatings while enhancing passivation in alkaline solutions with nanocrystalline Ni coatings by refining grain size and reducing diffusion thickness [100]. Ren et al. developed a durable superhydrophobic N-B/WC@MoS_2_ composite coating via US-mediated electrodeposition, thereby improving incorporation and performance [101]. At an optimal power of 270 W, the coating forms a dense, cauliflower-like nanostructure that improves hydrophobicity and corrosion resistance, with an enhanced *R*_p_ (3.56 Ω·cm^2^) and reduced corrosion rates. Corrosion pit development is delayed by the WC@MoS_2_ matrix, which provides passive shielding and ingress blocking. The synthesis of WC@MoS_2_ involves a 1:1 molar ratio mixture, thermal treatment, and processing to improve dispersion and reduce energy consumption, with US cavitation to enhance mass transfer and to incorporate it into the Ni-B matrix [102]. Future research should aim to integrate US transducers into continuous electroplating tanks, as most studies to date focus on laboratory-scale systems. Key factors to optimize include electrolyte flow, bath temperature, transducer fouling, and US activity during extended operations. Implementing these continuous US-assisted electroplating systems could enhance corrosion resistance, produce finer grain structures, and improve the uniformity of industrial metal coatings.

### 4.2 US in anticorrosive organic and polymer coatings

The US plays a vital role in enhancing organic and polymer-based anticorrosive coatings through US irradiation [103], [104], [105]. This technology improves dispersion, interfacial bonding, and structural uniformity by utilizing localized energy from acoustic cavitation, thereby accelerating mass transfer and promoting polymerization. It effectively mixes and breaks up agglomerated nanoparticles, leading to uniform distribution of active agents, finer filler exfoliation, and denser protective films that reduce porosity. Moreso, US irradiation enhances surface cleaning and activation, resulting in stronger adhesion [106]. Table SI-4 summarizes major reports on US-mediated organic and polymer coatings. The inspection of the data shows that across different coating systems, the US plays a primary role in homogenization, dispersion, exfoliation, in situ polymerization, surface activation, and acceleration of electrodeposition. In these coating systems, the increase in corrosion resistance is mainly due to reduced porosity, enhanced encapsulation, improved passivation, and interfacial adhesion. The tortuous or labyrinthine effects also play a significant role.

**Fig. 7:**
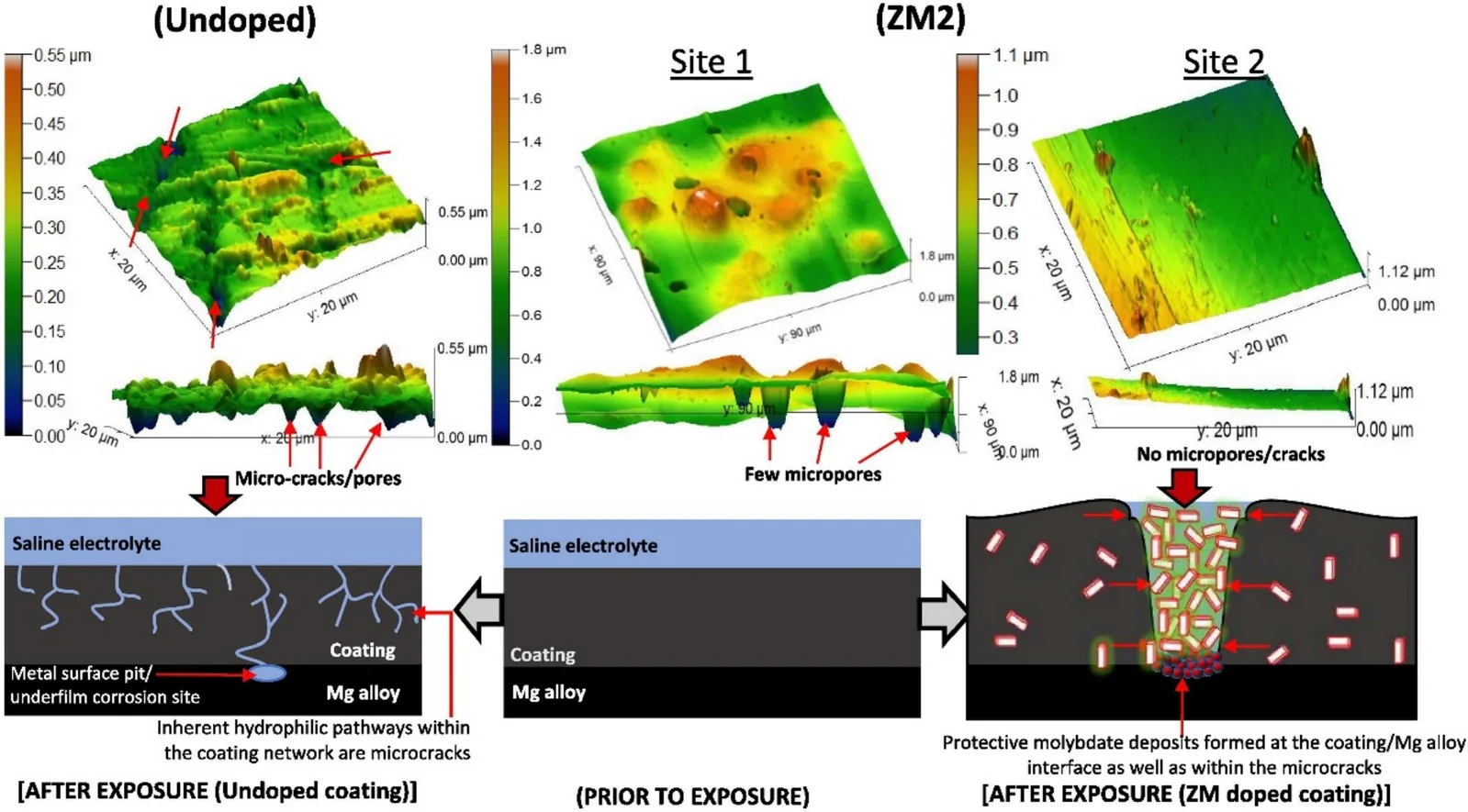
Schematic illustration of the mechanism of corrosion protection in EP/PDMS coating systems and the role of ZnMoO_4_ and Mo in corrosive species diffusion and protective precipitation [114]. (Reproduced with permission, © 2018 Elsevier Ltd. All rights reserved).”

In EP-clay nanocomposite systems, corrosion resistance improves due to reduced ingress of corrosive species [107], [108], [109]. US irradiation enhances polymerization in ZnO-PBMA and ZnO-PMMA hybrid systems, improving particle distribution and properties [110], [111]. The US process also benefits the crystal morphology in Zn-ferrocene and PPy-Zn coatings [112], [113]. Eduok and Szpunar reported that US-assisted synthesis of ZnMoO_4_ nanocrystals significantly boosts corrosion resistance in EP/PDMS coatings on Mg alloys in 3.5% NaCl, with optimal performance at 2 wt.% [114]. Fig. 7 shows the mechanism of corrosion protection in EP/PDMS coating systems. Higher concentrations lead to deamination and wider ingress pathways. ZnMoO_4_ acts as a filler, creating barriers against corrosive species, while molybdenum serves as a chemical inhibitor. US also enhances polymer-metal bond formation in coatings such as OFTES silane and PDA, thereby improving protective effectiveness [115]. Various studies support the dispersion of particles and their anticorrosive properties [113], [116].

### 4.3 US in anticorrosive sol-gel and ceramic coatings

The US also contributes significantly to the development of sol-gel and ceramic coatings, particularly through advances in mass transfer, induced acoustic cavitation, and controlled growth and nucleation at the micro- and nanoscale [[117], [118], [119]]. The localized heating, pressure, and shock waves from acoustic cavitation enhance hydrolysis and condensation sol-gel reactions, which accelerate ceramic suspension and particle dispersion. Ultrasound irradiation improves particle-size distribution, colloidal stability, and coating density, thereby extending corrosion protection [120], [121]. The role of US irradiation in sol-gel coatings enhances hydrolysis and condensation reactions, promoting M-O-M network formation while preventing uncontrolled cluster growth. In Si-based sol-gel studies, US-fabricated sols exhibit more uniform, homogeneous, and finer particle morphologies than those from traditional synthesis methods[122], [123]. Wang et al. developed a superhydrophobic CaCO_3_-based coating on Mg-Nd using US-assisted chemical conversion and stearic acid modification [122]. The study found that US irradiation enhances the coating's compactness, corrosion resistance, and uniformity in chloride-containing electrolytes, providing superior self-healing and anticorrosion properties compared to conventional water-bath treatments. In ceramic coatings, US aids in improving suspension homogeneity and controlling particle nucleation during precipitation. Specifically, in CaP and CaCO3 systems, US irradiation modifies particle size and crystal morphology, resulting in a better distribution of crystallites than under static or water-bath conditions, as evidenced by comparative micrographs [124], [125].

Such US-mediated microstructural refinement improves coating density, reduces surface defects, and retards the ingress of corrosive species. These studies also suggest improvements in mechanical interlocking via US irradiation. The US also uses sol-gel/ceramic composite coatings, where nanoparticles are incorporated into an inorganic or organosilane matrix [124], [125], [126]. In these coating systems, US irradiation ensures uniform dispersion of nanoparticles, enhancing physical protection and torturous effects. The US irradiation also helps reduce microcracking during coating drying, thereby improving mechanical stability. Therefore, the overall central role of US irradiation in anticorrosive ceramic and sol-gel coatings relies on its tendency to: (i) control crystallization and nucleation kinetics; (ii) intensify hydrolysis-condensation reactions; (iii) enhance coating density and homogeneity; (iv) minimize nanoparticles agglomeration, and (v) improve surface adhesion. These combined effects provide anticorrosive coatings with smoother morphology, lower porosity, and enhanced corrosion resistance.

### 4.4 US in self-healing anticorrosive coatings

**Fig. 8:**
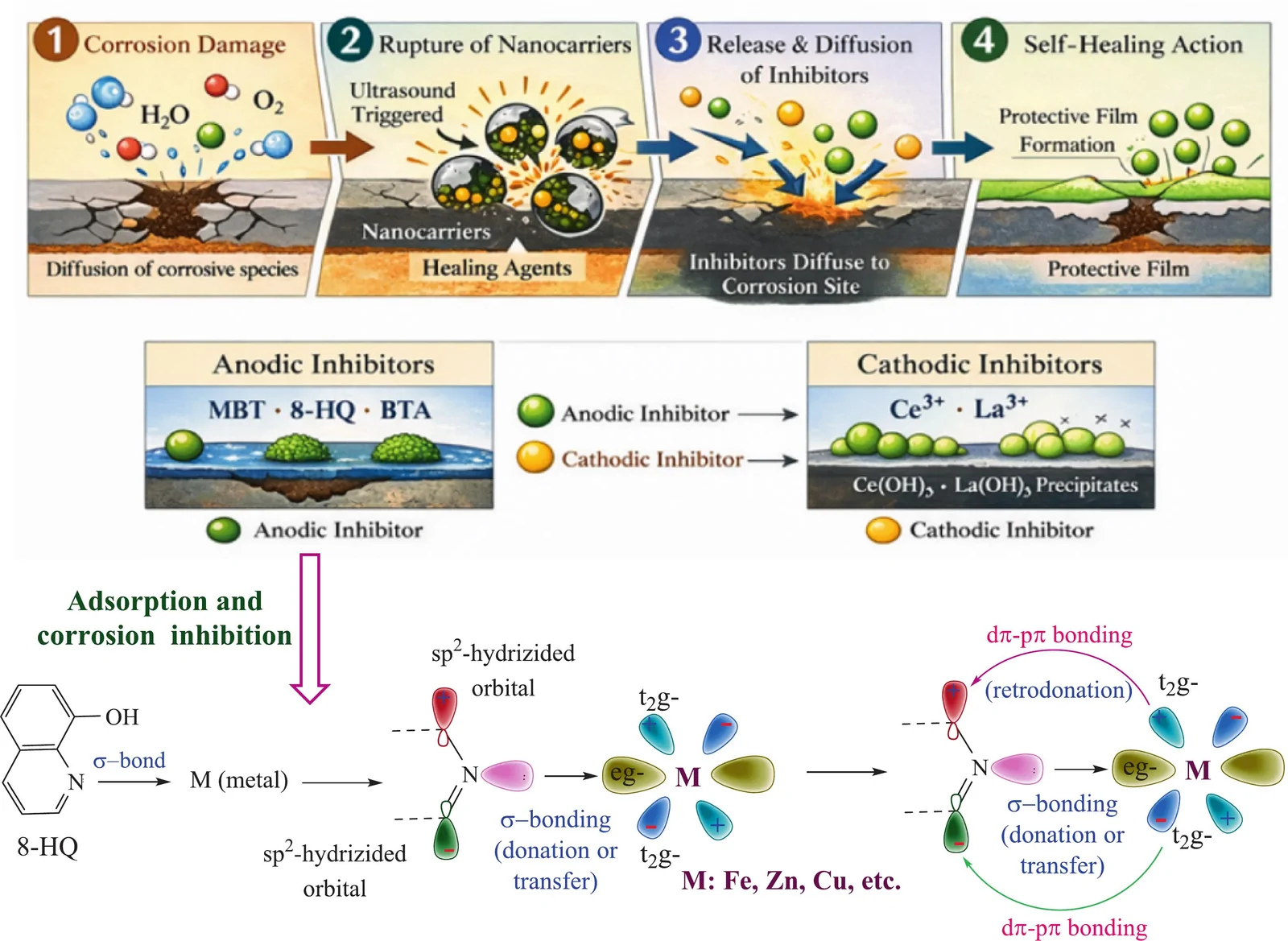
Schematic illustration of different steps in self-healing coatings and the mechanism of anodic and cathodic reactions inhibition.

Recently, self-healing coatings have emerged as a potential tool in industrial corrosion protection [127], [128]. These coatings are specifically designed to revert to their original form and restore functionality after corrosion, thereby improving the reliability and service life of metallic structures. These coatings incorporate active components (corrosion inhibitors or healing agents), typically encapsulated in micro- or nano-containers, called nanocarriers. The active agents remain inactive or in a dormant phase until corrosion-related damage occurs [127], [129] The nanocontainers rupture in response to localized corrosion and release the active components into the damaged areas, which in turn heal the defects and restore the barrier properties either through retarding the corrosion through forming corrosion protective films or retarding (or slowing) the diffusion of corrosive species through labyrinth or tortuous effects [130], [131]. In such coating systems, the healing process is mainly accomplished by the following order (Fig. 8): (i) corrosion-related damage and diffusion of corrosive species (electrolyte, ions, or gases), (ii) rupturing of nanocarriers containing active agents, (iii) release of active agents and their transfer (diffusion) to corrosion sites, and (iv) adsorption, precipitation or passive layer formation by active agents, retarding anodic, cathodic or both reactions.

Organic corrosion inhibitors, including 2-mercaptobenzimidazole (MBT), 8-hydroxyquinoline (8-HQ), and benzotriazole (BTA), coordinate to metal surfaces or metal cations to form protective hydrophobic films, most of which chelate and inhibit metallic dissolution [132], [133], [134]. They mainly serve as anodic corrosion inhibitors. On the other hand, rare-earth cations, such as Ce^3+^ and La^3+^, form insoluble precipitates in cathodic regions, primarily providing cathodic protection [135], [136], [137]. Recently, advanced self-healing systems have utilized encapsulated nanocarriers containing anodic and cathodic corrosion inhibitors. US irradiation enhances the dispersion, formation, and corrosion protection of these nanocarriers, improving the reliability of self-healing systems. Studies have demonstrated that US can also characterize self-healing coating systems [138]. Notably, Koh et al. fabricated polyurethane microcapsules containing oleate- and triazole-based inhibitors via interfacial polymerization and integrated them into primer coatings for on-demand corrosion protection [139].

The microcapsules were characterized using TGA, salt spray testing, FTIR, and SEM. The US was used for emulsification during microcapsule synthesis. The outcomes showed that, compared with mechanical agitation or shaking, US-mediated synthesis (20 kHz, 70% amplitude) produced more uniform microcapsules, finer (12-20 μm), and smaller, with a narrower size distribution. The shear forces and stringer cavitation effects improve microcapsule homogeneity and the controlled formation of the shell. Another study documented the US-mediated synthesis of dibutyl phthalate (DBP) nanocapsules via in situ polymerization with a UF (urea-formaldehyde) shell [140], followed by their incorporation into the EP coatings. Bagale and coworkers developed US-assisted neem oil (*Azadirachta indica*) nanocapsules via in situ polymerization of urea (U) and formaldehyde (F). They assessed their self-healing potential in EP polyamide coatings [141]. The authors employed US (120 W, 20 kHz) for emulsification and polymerization to develop nanocapsules with a diameter of nearly 65 nm. The US irradiation reduced agglomeration, enhanced dispersion stability, and increased corrosion resistance by reducing current density and increasing R_ct_. Other studies reported similar findings [[142], [143]]. The results showed that US irradiation greatly enhanced corrosion resistance by improving coating uniformity and air trapping. Therefore, in self-healing systems, US plays the following roles: (i) reduces micro- or nanocapsule size and involves cavitation-assisted emulsification; (ii) enhances micro- or nanocapsule distribution and dispersion in coating matrix; (iii) optimizes nanocarrier shell strength and stability, controlling on-demand rupture and release processes; and (iv) provides a non-destructive evaluation of structural uniformity and coating thickness.

## 5 Mechanisms of corrosion resistance enhancement

**Fig. 9:**
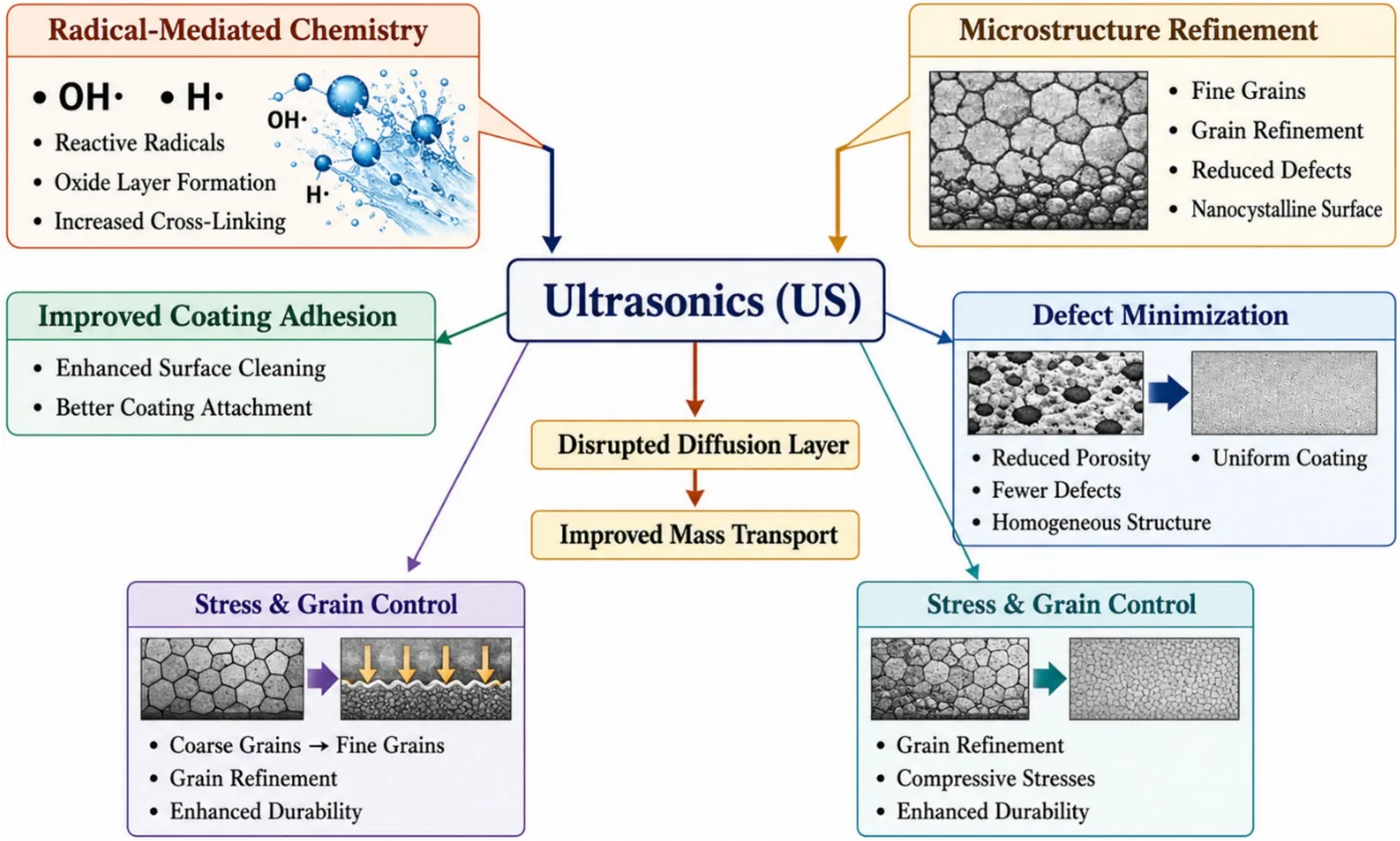
Schematics showing the role of different US-based strategies in corrosion resistance enhancement of metallic substrates.

The US also plays a central role in enhancing the corrosion resistance of metallic materials by altering surface and microstructural chemistries (Fig. 9). Acoustic cavitation produces enormous localized heat and pressure, along with microjets and shock waves near the solid surface [144]. These physicochemical effects impact the corrosion resistance of metallic surfaces. US enhances this resistance by promoting radical-mediated surface reactions, modifying diffusion-layer properties, improving coating adhesion, and refining microstructure through controlled methods. A key aspect is altering diffusion layer dynamics and mass transport, which depend on the transport of aggressive ions, water, and oxygen. US disrupts the hydrodynamic boundary layer through acoustic cavitation microjets and streaming, thereby significantly increasing the mass transport coefficient [76]. This effect appears to increase the C_R_ during the initial stage via uniform electrochemical mechanisms, thereby reducing the risk of localized corrosion, such as crevice and pitting corrosion. US modifies cathodic reactions mainly through enhanced diffusion-layer thinning, convection, and improved mass transport. Depending on the operating conditions and electrochemical system, these properties may increase cathodic limiting currents or modify reaction kinetics rather than uniformly conquering cathodic processes. Such coatings are less porous and denser, reducing the ingress of electrolyte and corrosive species.

The US is also known for its role in radical-mediated chemistry through sonochemical effects. The bubble collapsed during an acoustic cavitation, producing highly reactive hydrogen (^•^H) and hydroxyl (^•^OH) radicals via homolytic cleavage of the OH-H bond of water [145], [146], [147]. Highly reactive radicals can initiate polymerization, crosslinking, and oxidation reactions on metal surfaces. In sol-gel processing, US treatment enhances the hydrolysis-condensation reactions of metal alkoxides, resulting in homogeneous oxide frameworks with fewer structural defects and greater density [148], [149]. The compact oxide layers act as passive films that protect against corrosion by slowing anodic dissolution and preventing pitting corrosion. US irradiation enhances polymer-based anticorrosive coatings by increasing cross-linking density and promoting free-radical polymerization, thereby reducing the ingress of corrosion agents and improving barrier protection.

US treatment also provides microstructural and defect control [150], [151]. Microcracks, inclusions, porosity, and second-phase particles are initial sites for crevice and pitting corrosion. US-mediated cavitation reduces surface defects in coating deposition by breaking up particle agglomeration and dislodging trapped gas bubbles, especially in composite electroplating systems [152]. The US improved nanoparticle distribution within coatings, resulting in a more homogeneous microstructure with reduced surface heterogeneity and reduced galvanic coupling. Additionally, US irradiation enhances grain refinement in solidification and molten-metal processes by breaking dendrites and promoting heterogeneous nucleation [153], [154]. Grain refinement enhances corrosion resistance by enabling more uniform, superior passive films and minimizing microsegregation. The US treatment can also alter the surface nanocrystals by inducing plastic deformation, generating a nanocrystalline surface layer and compressive residual stresses [155], [156], [157]. Compressive residual stresses generally counteract tensile stresses (which drive crack propagation) and mitigate stress-corrosion cracking. Therefore, the combined effects of grain refinement, reduced porosity, and compressive stress significantly improve corrosion resistance.

The enhancement of corrosion resistance via irradiation is linked to improved coating adhesion, achieved through effective surface cleaning that removes contaminants, thereby enhancing wettability and interfacial bonding [158]. Despite advancements in US-assisted corrosion science, challenges persist, such as a lack of a unified mechanistic framework and insufficient exploration of long-term performance in realistic conditions [159], [160]. Scale-up issues, including acoustic field uniformity and reactor desing, also impede industrial application. Effective management of the acoustic field and reactor optimization are necessary for reproducible and efficient processing, alongside integration of regular calibration and modular designs to improve long-term operation and reduce maintenance costs. Although most of the existing US-assisted corrosion studies have been conducted under laboratory conditions using bath- or probe-type US reactors, several US technologies have already stretched industrial maturity in related fields, including US emulsification, leaning, surface modification, sonoelectroplating, and US extraction. These industrial applications indicate that US processing is technically feasible, although the corrosion-based industrial applications remain relatively limited. Despite significant advancements in laboratory-scale studies, pilot-scale or full industrial implementations of US-assisted corrosion mitigation remain limited. Therefore, quantitative information on reactor capacity, energy consumption, throughput, sonotrode wear, maintenance requirements, transducer lifetime, and technology readiness is infrequently reported in the literature. Consequently, the industrial debate presented in this review should be viewed as prospective, outlining engineering considerations and future pathways for industrial translation rather than as established industrial practice. Therefore, the primary challenge is not the scalability of US itself but rather the translation of laboratory-based corrosion mechanisms into reliable, continuous industrial processes that maintain uniform cavitation intensity across large process volumes.

Scale-up of US technologies for industrial applications necessitates transitioning from small-batch reactors to continuous-flow systems with synchronized transducers. Challenges in industrial reactors include standing-wave formation and variable cavitation intensity, which complicate processes such as nanoparticle dispersion and coating deposition. To ensure consistent performance, advanced reactor engineering, acoustic field modeling, and continuous liquid recirculation are critical. Economic considerations must balance corrosion-mitigation efficiency with operational costs. A life-cycle analysis is recommended before widespread industrial implementation. Initial lab testing should focus on the impact of cavitation on corrosion resistance, followed by validation in pilot-scale reactors. Further development should refine reactor desing and establish standardized operating procedures for applications like passivation and electroplating. Additionally, a lack of standardized reporting of US processing conditions has been identified, emphasizing the need for comprehensive documentation of experimental variables to enhance reproducibility and promote adoption in industry.

To enable meaningful comparisons and facilitate reproducibility across independent studies, a minimum set of calibration and US processing parameters should be measured and reported in each study. Currently, several reports provide only the generator power and nominal US frequency, while numerous variables that directly influence electrochemical behavior and cavitation intensity are often omitted. Some major variables include actual delivered power, acoustic power density, probe immersion depth, specimen position, transducer geometry, solution temperature, dissolved gas concentration, reactor dimensions, and quantitative characterization of cavitation. As nominal power does not adequately represent the acoustic energy delivered to the electrolyte, standardized reporting of these parameters is mandatory for interpreting corrosion data and comparing results across laboratories. Table SI-5 presents the recommended minimum reporting parameters for US-assisted corrosion studies, along with suggested measuring methods and their significance. In addition to reporting US operating parameters, complementary cavitation characterization approaches such as cavitometry, chemical dosimetry, sonochemiluminescence, and foil-erosion testing should be reported where applicable to assess cavitation activity. Moreover, hydrophone testing may also be valuable for acoustic field characterization. Measurements under cavitation should be interpreted cautiously, as bubble clouds can perturb the acoustic field and affect the measured acoustic pressure. The adoption of these minimum reporting data will substantially enhance reproducibility, facilitate comparisons of data across different studies (laboratories), and accelerate laboratory-to-industrial translation. More significantly, comprehensive and systematic reporting will also allow researchers to distinguish true material-dependent effects from changes arising solely from differences in reactor configuration or acoustic field characteristics.

## 6 Critical analysis of ultrasonic effects across corrosion systems

Although the literature consistently demonstrates that US can enhance corrosion resistance by improving mass transport, nanoparticle dispersion, coating densification, and grain refinement, these improvements are highly system-dependent. Acoustic cavitation alters coating growth and transport phenomena through acoustic steaming, shock waves, and microjet impregnation. In contrast, the ultimate corrosion response depends strongly on US frequency, reactor geometry, power density, and exposure conditions [27], [161]. Careful observation of the published literature shows that corrosion resistance rarely increases monotonically with US intensity. Instead, several studies report an optimum operating region (range) in which moderate cavitation produces remarkable enhancement in electrochemical performance and coating quality. In contrast, excessive cavitation causes localized corrosion, surface erosion, microcracking, and disruption of the passive film. This observation suggests that the apparently contradictory findings reported in the literature are better interpreted as arising from different cavitation regimes rather than genuine inconsistencies [35], [162].

**Fig. 10:**
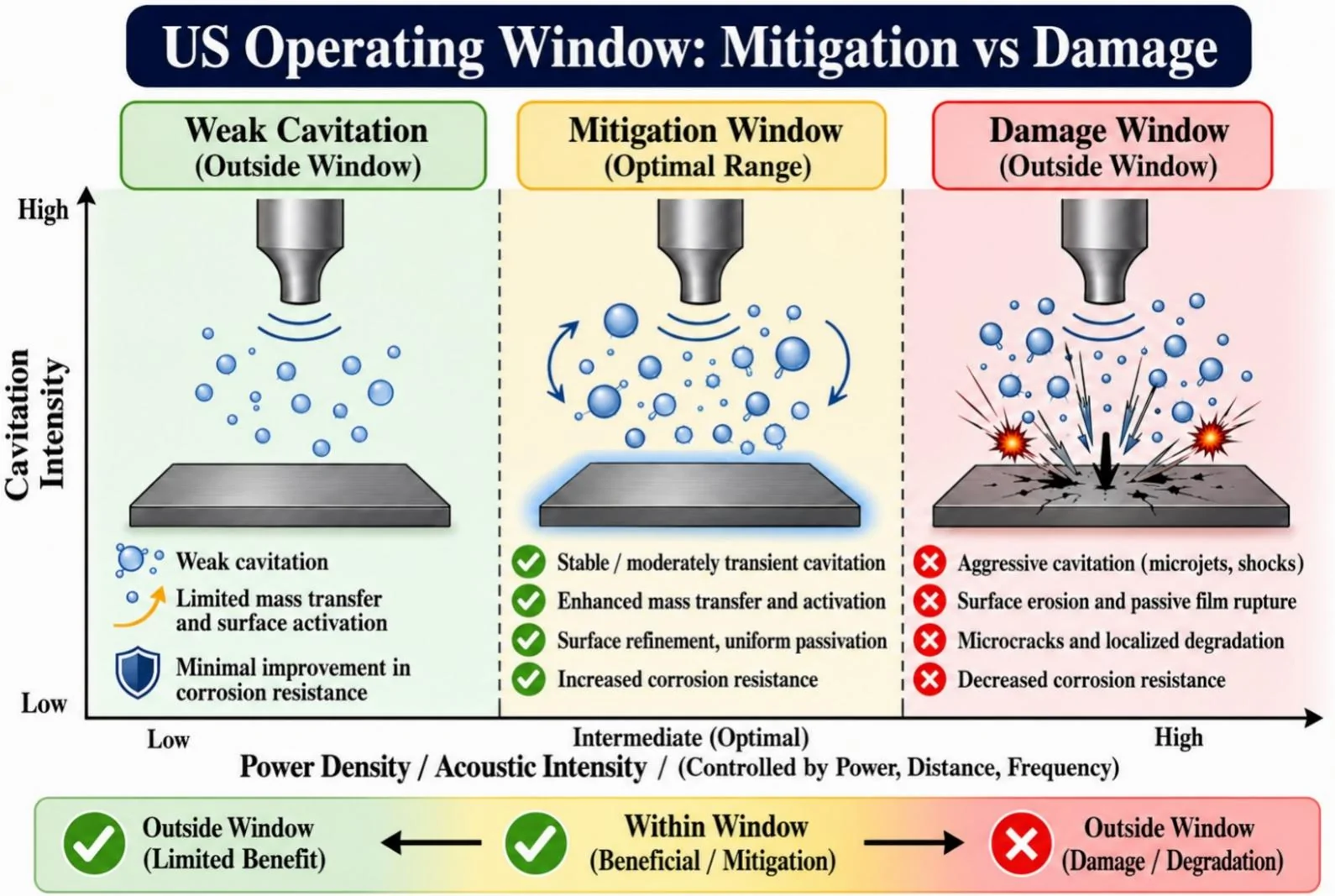
Conceptual framework showing proposed shift from weak cavitation to corrosion-protection and cavitation-damage regions as a function of cavitation intensity. The figure is intended as a qualitative representation and does not define quantitative operating boundaries.

To further rationalize these contrasting effects, a concept of operating-window can be used to differentiate US-assisted corrosion mitigation from cavitation-mediated damage. At excessive substrate-horn distance or at low power density, cavitation is weak and generates only limited acoustic steaming. Under these circumstances, surface activation, mass-transport enhancement, or coating densification have modest effects. At intermediate power strength (density), controlled substrate distance, and moderate frequency, stable or moderately transient cavitation accelerates diffusion-layer thinning, hydrogen bubble removal, grain refinement, nanoparticle dispersion, and uniform passivation, increasing overall corrosion resistance. This region may be considered as a “mitigation window”. Conversely, at relatively high-power density, very small substrate-horn distance, or low frequency, aggressive near-boundary cavitation dominates, generating shock-wave loading, microjet impact, localized erosion, passive film rupture, and microcrack formation. This region can be represented as a “damage window”. Fig. 10 presents a conceptual framework illustrating the proposed transition among weak cavitation, corrosion mitigation, and cavitation-damage regimes. It is intended as a qualitative interpretation of the reported literature rather than a quantitative operating map. weak cavitation to corrosion-mitigation and cavitation-damage regions as a function of cavitation intensity. The exact boundary between the mitigation and damage windows is material-specific. For example, several stainless steels (SS) generally exhibit greater resistance to cavitation-driven degradation than carbon steels (CS) due to their repassivation capability. However, the response varies depending upon microstructure, alloy composition, electrolyte chemistry, surface conditions, and repassivation kinetics [163], [164]. This suggests that the operating window should be interpreted as a conceptual guide rather than a universal design rule.

Another significant observation is that the major corrosion-resistant enhancement mechanism lies strongly in the material system under investigation. Polymeric and nanocomposite-based coatings benefit from reduced porosity, improved interfacial adhesion, and enhanced nanoparticle dispersion. In contrast, metallic electrodeposition mainly benefits from grain refinement, hydrogen bubble removal, and accelerated ion transport. On the other hand, passivating alloys benefit from enhanced passive film uniformity and accelerated depassivation-repassivation cycles. Therefore, because the governing mechanisms differ substantially, the qualitative comparison among various material systems should be interpreted with caution [162], [165]. Lastly, the literature lacks systematic multi-parameter optimization. In most of the published studies, only one US parameter, mainly acoustic power, varies, while keeping other parameters, including reactor geometry, liquid height, frequency, duty cycle, and acoustic field distribution, unchanged. Since the intensity of cavitation depends on these parameters, many previously reported optimal conditions apply only to a specific reaction, i.e., are not universally applicable. Therefore, future studies should use statistically designed experimental methodologies, along with quantitative cavitation characterization, to establish transferable operating windows [27], [162].

The ongoing discussion shows that considerable progress has been made in understanding of US-assisted corrosion phenomena; however, several scientific and engineering challenges continue to limit their industrial implications. The future research should prioritize: (a) quantitative characterization of cavitation intensity through hydrophone and calorimetry mapping; (b) development and establishment of standardized US testing protocols; (c) optimization of continuous flow US reactors; (d) pilot-scale validation under realistic conditions; (e) long-term durability measurement under cyclic service conditions; (f) coupling of computational modeling with reactor desing and (g) comprehensive life-cycle and techno-economic assessment. Effective addressing of these challenges will significantly accelerate the translation of laboratory-scale discoveries into commercially viable corrosion mitigation technologies.

## 7 Conclusion, challenges, and outlooks

The present review provides that sonochemical technologies have emerged as versatile and powerful strategies for advancing corrosion science and engineering. Acoustic cavitation produces coupled sonochemical and sonophysical effects that greatly affect the surface microstructure, interfacial electrochemistry, and mass transfer, all of which contribute to corrosion. The literature shows that though uncontrolled cavitation may boost surface degradation, optimized US conditions enhance surface activation, nanoparticle dispersion, passivation uniformity and effectiveness, and coating density. US treatment also enables the efficient, eco-friendly synthesis of corrosion inhibitors with enhanced performance, including bio-based and sustainable corrosion inhibitor systems and advanced materials such as grafted polymers and nanocomposite coatings. The US is highlighted as a multifunctional tool for advanced corrosion mitigation approaches, although its sustainability and environmental advantages require verification through quantitative techno-economic and life-cycle assessment (LCA) studies.

Although the use of sonochemical technologies in corrosion science and engineering offers several advantages, shortcomings limit their broader applications. The highly nonlinear dependence of corrosion behavior on US parameters, such as power, reactor desing, exposure time, and frequency, complicates reproducibility and process optimization. Moreover, excessive cavitation can cause adverse effects, including microstructural damage, surface erosion, and coating degradation. Owing to energy inefficiencies and non-uniform acoustic field distribution, scaling up these systems remains challenging, especially for large systems. The universal design and subsequent application are also greatly challenged by material-specific responses. The industrial adoption of these systems is also limited by a lack of long-term performance data and by their high energy consumption. Lastly, the highly complex coupling between sonochemical and sonophysical effects requires deeper mechanistic insight. These challenges lead to various outlooks for the future. Future studies should focus on optimizing US processing through advanced reactor desing, such as flow-through and multi-transducer systems, to enable scalable applications. Future advancements should also consider integrating US into the synthesis and development of smart, self-healing materials, including encapsulated, stimuli-responsive inhibitor systems, offering promising pathways towards sustainable, on-demand corrosion protection. As the US-mediated synthesis of self-healing systems, corrosion inhibitors, nanocomposites, and grafted polymers aligns with the aims and objectives of sustainability in materials development, this should be explored. Future research should also focus on evaluating solvent use, waste production, energy consumption, process mass intensity, and techno-economic or life-cycle assessment performance to substantiate sustainability claims. These aspects should be explored in future investigations. The development of in situ US monitoring tools and standardized protocols will be critical for industrial translation. From a practical point of view, effective use of US-assisted corrosion mitigation requires careful optimization rather than maximizing US intensity. Generally, the most favorable balance between reduced mechanical damage and improved electrochemical transport is achieved at moderate cavitation levels. In contrast, excessive cavitation may cause adverse effects, compromising passive-film stability and coating integrity. Therefore, to better translate laboratory-scale testing to industrial practice, future studies should focus on standardized experimental protocols, statistically designed parameter optimization, quantitative calibration, and prolonged validation under realistic conditions.

## CRediT authorship contribution statement

**Chandrabhan Verma:** Writing – original draft, Supervision, Conceptualization. **Anu Radha Pathania:** Writing – original draft, Resources. **Akram AlFantazi:** Writing – review & editing, Visualization, Validation, Supervision, Funding acquisition.

## Declaration of competing interest

The authors declare that they have no known competing financial interests or personal relationships that could have appeared to influence the work reported in this paper.

## Appendix A Supplementary data

Supplementary Data 1 Table SI-1 to SI- 5 & Figure SI-1 to SI-6.
