# Supplementary material for: Sonochemical technologies in corrosion science and engineering: from laboratory studies to industrial perspectives
Source: Ultrason Sonochem. 2026 Aug 25;133:108024. doi: 10.1016/j.ultsonch.2026.108024 (PMC13544107; doi:10.1016/j.ultsonch.2026.108024)
Supplement: Supplementary file 1 [file mmc1.docx]

**Supporting information (SI)**

**Sonochemical Technologies in Corrosion Science and Engineering: From Laboratory Studies to Industrial Perspectives**

Chandrabhan Verma^1,2^*, Anu Radha Pathania^3^, and Akram AlFantazi^1,2^

^1^Department of Chemical and Petroleum Engineering, Khalifa University of Science and Technology, P.O. Box 127788 Abu Dhabi, United Arab Emirates

^2^Emirates Nuclear Technology Center (ENTC), Khalifa University of Science and Technology, Abu Dhabi, 127788, United Arab Emirates

^3^Department of Chemical Sciences, Parul Institute of Applied Sciences, Faculty of Applied Sciences, Parul University, Waghodia, Vadodara, Gujarat, 391760, India

**Corresponding Author’s details**: [chandraverma.rs.apc@itbhu.ac.in](mailto:chandraverma.rs.apc@itbhu.ac.in) (CV); Department of Chemical and Petroleum Engineering, Khalifa University of Science and Technology, P.O. Box 127788 Abu Dhabi, United Arab Emirates

**Author’s Credit Note**: CV and ARP contribute equally.

**Table 1**: Summary of some major reports on inhibitory potential of US-assisted corrosion inhibitors for steel (MS or CS) alloys in acidic (1 M or 15% HCl) solutions.

| S/N | Structure, Name &abbreviation | Electrolyte/  Metal | Best %IE & Conc. | Nature of adsorption | | Order of efficiency | Synthesis | | Reference |
| --- | --- | --- | --- | --- | --- | --- | --- | --- | --- |
|  |  |  |  | PDP | Isotherm |  | Reaction time | Yield |  |
| 1 |  | 1M HCl/ MS | 97.7%/ 10 ppm | Mixed type | Langmuir | 97.7%/ 10 ppm | 30-35 min | ~84% | [44] |
| 2 |  | 1M HCl/ CS | 97.6%/ 10^-2^ M | Mixed type | Langmuir | MA2 (97.6%) > MA1 (94.6%) | 5h | 79% | [46] |
| 3 |  | 1M HCl/ MS | 95.2%/ 100 ppm (Ap-5) | Mixed type (slight cathodic) | Langmuir | AP-5 (95.23%) > AP-4 (94.4%) > AP-3 (94.0%) > AP-2 (91.10%) > AP-1 (90.5%) > | 51-70 min | 86-90% | [47] |
| 4 |  | 1M HCl/ MS | 95.23%/ 300 ppm (INH-3) | Mixed type | Langmuir | INH-3 (95.23%) > INH-2 (93.33%) > INH-1 (91.90%). | 20-25 min | NR* | [45] |
| 5 |  | 1M HCl/ MS | 96.63%/ 100 ppm (PP-1) | Mixed type | Langmuir | PP-1 (95.23%) > PP-2 (93.61%) > PP-3 (91.38%). | NR* | NR* | [48] |
| 6 |  | 15% HCl/ N80 steel | 93.2%/ 800 ppm (TZ-3) | Mixed type | Langmuir | TZ-3 (93.2%) > TZ-1 (90.6%) > TZ-2 (86.0%). | 3-5 min | NR* | [49] |
| 7 |  | 1M HCl/ MS | 99.93%/ 0.06M (IND-2) | Mixed type | Langmuir | IND-1 (99.93%) > IND-2 (99.69%) > IND-3 (98.12%). | 3-5 min | NR* | [50] |

*NR: Not reported

**Table SI-2**: A summary of some major reports on UEE and UEE plant extract-based corrosion inhibitors.

| S/N | Biomass/ Plant | Electrolyte/  Metal | %IE/ Conc. | Nature of inhibition | | Contribution of the US irradiation | Ref. |
| --- | --- | --- | --- | --- | --- | --- | --- |
|  |  |  |  | PDP | Isotherm |  |  |
| 1 | Acorns' tannins (*Quercus* spp.) | 3.5% NaCl/ MS | 86.0%/140 ppm | Mixed type | NR | US-assisted enzymatic extraction | [66] |
| 2 | *Catharanthus roseus* (root and stem extract) | 3.5% NaCl/ MS | 96% (stem) & 84% (root) (5 ppm) | Mixed type | Langmuir isotherm | US was used to obtain ethanolic extracts from the roots and stems of *C. roseus*. | [59] |
| 3 | Pectin from *Solanum lycopersicum* (tomato waste) | 1M HCl/ CS | 94%/ 2.0w/w% | Mixed type | NR | The US was used for the extraction of pectin from *Solanum lycopersicum* waste | [60] |
| 4 | *Abelmoschus esculentus* (Okra pectin) | 1M HCl/ ANSI 304 SS | 86.5%/ 2 g/L | Mixed type | Langmuir isotherm | Sweeping-frequency US pretreatment improves pectin modification and inhibitory efficiency. | [61] |
| 5 | *Platanus acerifolia* leaves extract | SCPS+0.5M NaCl /HPB235 C-steel | 96.657%/ 3.0% UAE | Mixed type | NR | UEE increases the extraction yield by 22-39% as compared to UAE | [62] |
| 6 | *Brassica napus* (Rapeseed meal) extract | 0.5M H_2_SO_4_ /CRS | 88.65% (RMUE) and 84.84% (200 ppm) | Cathodic type | Langmuir isotherm | RMRE showed relatively better extraction (15.2%) than RMUE (11.94%). The efficiency also followed the same trend. | [63] |
| 7 | *Nepeta cataria* (L. leaf) extract | 0.5M H_2_SO_4_ /Cu | 91.24%/ 800 ppm (25 °C) | Cathodic type | Langmuir isotherm | US enhances the release of phytochemicals from plant cells. | [64] |
| 8 | Tannin extract from *Acacia mearnsii* | 0.05 M NaCl/ AISI 1045 CS | 71.85%/ 1.0 g/L (neutral pH) | Mixed type | Langmuir isotherm | The US assisted in the purification of the tannin extract | [65] |

SCPS: Simulated concrete pore solution; CRS: Cold rolled steel; RMRE: Rapeseed meal reflux extract; RMUE: Rapeseed meal ultrasonic extract.

**Table SI-3**: A summary of US-based anticorrosive metallic coatings.

| S/N | Coating system | US parameter (range) | Effect of corrosion resistance | Main mechanism(s) | Microstructural effect(s) | Ref. |
| --- | --- | --- | --- | --- | --- | --- |
| 1 | AA2024 coatings via Ce/Al oxide | 57 W cm^-2^ (high intensity) US | Increased polarization resistance (R_p_) | Increase surface activation and Ce incorporation. | Enhance Ce-networking and induce strong interface bonding | [92] |
| 2 | Ni-MO/GO composite coatings | 60 kHz, 90 W cm^-2^ (electro-deposition) | Improved Rp (3.62 kΩ.cm^2^) and oxidation resistance. | Increase coating density and GO dispersion and reduce grain size. | Cavitation provides grain refinement and improves incorporation. | [93] |
| 3 | Multilayer Ni-coatings | US irradiation (intermittent) | Minimizes galvanic corrosion and increases uniform corrosion. | Multilayer coatings increase surface smoothness and reduce surface defects (pores). | The US provides pinhole reduction and alters grain orientation and distribution. | [94] |
| 4 | Zn-Co alloy coatings | 24 kHz, 38 W cm^-2^ | Enhanced R_ct_ (best at 20 W cm^-2^) | US refines the grains, increases mass transfer, and reduces diffusion layer thickness. | Enhance Co incorporation, dense and homogeneous morphology, and reduced dendrites. | [95] |
| 5 | MgF2 coatings on AZ31 Mg alloy | 14 and 28 kHz | Reduced i_corr_ and H_2_ evolution (0.32 mL/cm^2^ ) | High power increases compactness, as seen in pore-free MgF_2_ derived at 28 kHz. | The US increases interface density and decreases porosity. | [96] |
| 6 | Ni/GO composite coatings | 24 kHz, 20 W cm^-2^ (US-SC-CO_2_) | Enhances R_p_ (9073.8 Ω.cm^2^) of traditional Ni/GO and pure Ni coatings | Cavitation accelerated particle incorporation | The US improves the hardness and GO dispersion and reduces the grain size | [97] |
| 7 | Ni-W alloy coatings | Thermo-sonication (TS) at 40- 60 °C. | Enhances R_p_ (20.50 kΩ.cm^2^) | Reduced microcracks (surface imperfections) | US modifies alloy composition and improves diffusion. | [98] |
| 8 | Ni-W alloy coatings | 19 kHz probe, 100-400 W | Optimum R_p_ (23.42 kΩ.cm^2^) was derived at 200 W | Reduced erosion (power dependent) | 200-300 W of power provides uniform, smooth coatings and reduces grain size. | [24] |
| 9 | CaCO_3_/Mg(Al)O coating on AZ41 Mg alloy | US (40 kHz) assisted electro-deposition | Repassivation observed and C_R_ < 0.005 mm/y (0.6M NaCl) | The density of the CaCO_3_-Mg(Al)O double layer increases with increasing compactness. | Physical film compactness and passive film density increase. | [99] |
| 10 | Nanocrystalline Ni (NC) films | 30 kHz probe, 95-200 W | Enhance passivation and corrosion resistance (NaOH) | Increase mass transfer and decrease the thickness of the diffusion layer. | US reduces grain size and roughness and increases hardness. | [100] |
| 11 | Ni-B/WC@MoS_2_ (superhydrophobic) composite coatings | 20 kHz probe, 270 W | C_R_ was reduced three times, and R_p_ increased (3.56 kΩ.cm^2^) | Affect nanoparticle modification and dispersion. | The US improved surface hydrophobicity and barrier properties. | [101] |
| 12 | Ni-B/WC@MoS_2_ (superhydrophobic) composite coatings | US-assisted electro-deposition | Increase corrosion resistance in 3.5% NaCl | Affect nanoparticle modification and dispersion. | The US improved surface hydrophobicity and barrier properties and reduced surface roughness. | [102] |

**Table SI-4**: A summary of major reports on US-mediated organic and polymer coatings.

| S/N | Coating system | Role of US radiation | Corrosion performance | Main mechanisms | Ref. |
| --- | --- | --- | --- | --- | --- |
| 1 | EP-clay nanocomposite coatings | The US was used for clay exfoliation | Improved R_p_ at 3-5wt.% clay and minimized water ingress | Tortuous (or labyrinth effect) and improve dispersion | [107] |
| 2 | Polyurethane (PU)/organoclay composite coatings | The US was used for dispersion | Corrosion resistance and impedance were improved, especially by 3 wt.%. | Passivation (barrier) protection and uniform distribution of nanocomposites. | [108] |
| 3 | Water-based EP + nanoPANI | The US was used for homogenization | Corrosion resistance was improved significantly at 0.02 wt.% nanoPANI. | Passivation (barrier) protection and uniform distribution of nanocomposites. | [109] |
| 4 | ZnO-PBMA/polyaniline (PANI) coatings | The use was used for emulsion polymerization | Better corrosion resistance than traditional coating | Excellent encapsulation of ZnO and formation of dense barrier films. | [110] |
| 5 | ZnO-PMMA hybrid coatings | The US was used for the synthesis of a hybrid | C_R_ and i_corr_ were reduced significantly. | ZnO passive film was developed, and the nanoparticle dispersion was uniform. | [111] |
| 6 | Zn-Ferrocene composite  Coatings | The US was used with electro-deposition | Improved corrosion resistance and R_ct_ by two times | Improved crystal morphology and additive incorporation. | [112] |
| 7 | Polypyrrole-Zn (PPy) based coatings | The US was used with electro-deposition | Better protection and more dense protective films. | Enhanced mass transfer and homogeneous doping and distribution. | [113] |
| 8 | Mo-doped epoxy/PDMS nanocomposite coatings | The US was used for in-situ polymerization | Corrosion resistance and coating adhesion were improved. | Excellent filler encapsulation. | [114] |
| 9 | (1,1,2,2,3,3,4,4-octafluoropentyloxy)-propyltriethoxysilane (OFTES) coatings | The US was used in the silane deposition | Increased E_corr_, avoid water diffusion, and increase compactness. | Enhanced metal-Si-O bonding, reduced porosity, crack filling, and enhanced interfacial bonding. | [115] |
| 10 | Ti_3_C_2_T_x_-PDMS composite coatings | The US was used for monitoring purposes | Improve mechanical strength and corrosion resistance. | Photoacoustic and lamellar effects. | [117] |

**Table SI-5**: A summary of recommended reporting parameters for US-assisted corrosion studies, along with suggested measuring methods and their significance.

| S/N | Reporting Category | parameter | Recommended reporting | Significance of corrosion studies |
| --- | --- | --- | --- | --- |
| 1 | US Operating Conditions | Nominal electrical power (W) | Report generator output | Indicates instrument setting |
|  |  | Frequency (kHz) | Report nominal operating frequency | Governs bubble size, radical production, cavitation regimes, and collapse intensity. |
|  |  | Delivered acoustic power (W) | Report measured calorimetric power | Enables comparison between various laboratories and US systems |
|  |  | Volumetric power density (W L^-1^ or W cm^-3^) | Report calculated value based on probe area | Better descriptor of cavitation intensity |
|  |  | Duty cycle (%) | Specify pulsed or continuous operation | Strongly affects heating, cavitation stability, and coating quality. |
|  |  | Sonication time | Report total and effective sonication | Determines cavitation energy delivered to the system. |
| 2 | US Reactor Configuration | Reactor type | Bath, multi-transducer, probe, and flow-through | Reactor configuration strongly affects cavitation distribution |
|  |  | Probe diameter and immersion depth | Report dimensions and immersion distance | Influence acoustic-field distribution and cavitation behaviour |
|  |  | Dimension and solution volume | Report reactor geometry and liquid volume | Compares energy density among studies |
|  |  | Specimen position | Distance from transducer/probe | Local cavitation intensity varies with distance |
| 3 | Solution Conditions | Temperature | Report the initial and final temperature | Temperature alters electrochemical kinetics and cavitation threshold |
|  |  | Dissolved gas condition | Degassed, oxygenated, air-saturated, etc. | Cavitation intensity and bubble nucleation depend on dissolved gases. |
|  |  | Electrolyte composition | Report composition and concentration | Significant for the interpretation of corrosion mechanisms. |
| 4 | Cavitation Characterization | Calorimetry | Recommended whenever possible | Measures the actual acoustic power delivered to the system (liquid) |
|  |  | Hydrophone mapping | Recommended for detailed studies | Characterizes acoustic pressure field (where applicable); measurements under developed cavitation should be interpreted cautiously |
|  |  | Chemical dosimetry, sonochemiluminescence, cavitometry or foil-erosion testing | Report the method used and corresponding cavitation activity, where available. | Provides complementary assessment of cavitation activity and supports comparison and reproducibility of US-assisted corrosion studies. |
| 5 | Electrochemical Testing | Electrochemical protocol | Scab rate, EIS frequency range, OCP stabilization time, immersion duration | Enable comparison and reproducibility of corrosion performance |
|  |  | Replicates and uncertainty | Number of experimental and statistical analyses | Enhances the reliability of reported electrochemical corrosion data. |

**Figure SI-1.** A clear approach demonstrates how adjusting US parameters such as frequency, intensity, exposure duration, and reactor setup can control cavitation and enhance corrosion resistance.

**Figure SI-2.** Effect of US on diffusion boundary layer thickness. US irradiation reduces the diffusion layer and enhances the transport of corrosion inhibitor molecules toward the metal surface, thereby improving adsorption or surface coverage.

**Figure SI-3.** Mechanism of US enhancement of metallic passivation. Cavitation bubble collapse near the metal surface removes weak oxide patches and contaminants. At the same time, acoustic steaming enhances transport of oxygen and ions, promoting rapid repassivation and formation of a dense passive film.

**Figure SI-4.** Time-dependent evolution of passive film formation under US treatment. Initial surface cleaning is followed by rapid oxide growth, which eventually stabilizes a dense, protective passive layer.

**Figure SI-5.** US-assisted formation of encapsulated corrosion inhibitors. Cavitation-induced shear forces reduce droplet size during emulsification and promote uniform shell formation around inhibitor cores, resulting in a homogeneous distribution of microcapsules.

**Figure SI-6.** Time-dependent inhibitor release behavior in ultrasonically fabricated encapsulated systems. Controlled release provides sustained protection, while US activation can accelerate release when required.
